# Understanding drivers of stunting reduction in Nigeria from 2003 to 2018: a regression analysis

**DOI:** 10.1007/s12571-022-01279-8

**Published:** 2022-03-26

**Authors:** Olutayo Adeyemi, Mariama Toure, Namukolo Covic, Mara van den Bold, Nicholas Nisbett, Derek Headey

**Affiliations:** 1 Department of Human Nutrition and Dietetics, Faculty of Public Health, University of Ibadan, Ibadan, Nigeria; 2 International Food Policy Research Institute, Washington, DC USA; 3 Present Address: International Livestock Research Institute, Addis Ababa, Ethiopia; 4 Present Address: Clark University, Worcester, Massachusetts USA; 5 Institute of Development Studies, Brighton, UK

**Keywords:** Stunting, Nutrition, Multisectoral coordination, Scale-up, Africa

## Abstract

Nigeria is a high burden country for stunting. Stunting reduction has been slow and characterized by unequal progress across the 36 states and federal capital territory of the country. This study aimed to assess the changes in prevalence of stunting and growth determinants from 2003 to 2018, identify factors that predicted the change in stunting, and project future stunting prevalence if these predicted determinants improve. Trend and linear decomposition analyses of growth outcomes and determinants were conducted using 2003, 2008, 2013, and 2018 Nigeria Demographic and Health Survey data. Pooled data included 57,507 children 0 to 59 months old. Findings show that stunting and severe stunting significantly reduced from 43 to 37% and 23% to 17%, respectively (p < 0.001), between 2003 and 2018. Disturbingly, height-for-age z-scores at birth significantly decreased, indicating risks of potential future stunting increase. Improvements in nine stunting determinants (maternal body mass index, maternal height, ≥ 4 antenatal care visits, health facility delivery, reduced child illnesses, asset index, maternal education, paternal education, and preceding birth interval) predicted stunting reductions in children 0–59 months. Few of these nine determinants improved in subpopulations with limited stunting progress. Intra-sectoral and multisectoral coordination were potentially inadequate; 12% of children had received all of three selected health sector interventions along a continuum of care and 6% had received all of six selected multisector interventions. Forward looking projections suggest that increased efforts to improve the nine predictors of stunting change can reduce under-five stunting in Nigeria to ≤ 27% in the short term.

**Supplementary Information:**

The online version contains supplementary material available at 10.1007/s12571-022-01279-8.

## Introduction

Nigeria is a high burden country for chronic undernutrition. There were more than 11.9 million stunted children under 5 years old in Nigeria in 2020 – about 8% of the world’s stunted children and more than half of stunted children in West Africa (UNICEF et al., 2021). While there has been a reduction in prevalence of stunting over the past few decades, from 42% in 2003 to 37% in 2018, progress has been slow, and Nigeria is not on track to meet global stunting reduction targets (Development Initiatives, 2017). In addition, progress has not been uniform across the country. Some states have experienced reductions in stunting, while others have seen stunting increase (NPC & ICF, 2019; NPC & Macro, 2009).

**Fig. 1:**
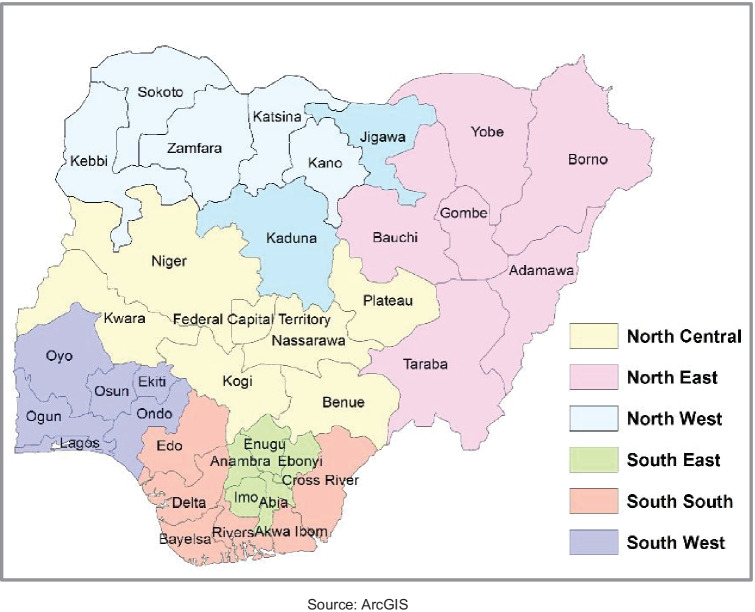
Map of Nigeria showing 6 Geopolitical Zones and highlighting Jigawa and Kaduna States in the North West Zone

All nutrition-related sectors, including health, education, agriculture, and sanitation, are on a constitutional concurrent list in Nigeria. For such sectors, responsibilities are shared among the government at federal, state, and local government area (LGA) levels, and each level of government has implementation autonomy. Thus, each of the 36 states and federal capital territory (FCT), Abuja, as well as each of the 774 LGAs across the country can decide on whether or not they take action on issues related to these sectors, what actions to take, and how much they choose to spend (Akindele et al., 2002; Khemani, 2001). In states that have seen a reduction in stunting prevalence, it is not clear which determinant factors may be associated with the observed reductions. Indeed, this is even true at more aggregated geographies; the 36 states and FCT in Nigeria are aggregated into 6 geopolitical zones (Fig. 1) that have also seen apparent differences in stunting trends (NPC & ICF, 2019; NPC & Macro, 2009).

Given the poor economic, education and health outcomes associated with stunting (Hoddinott et al., 2013; Shekar et al., 2014), it is important that the factors driving (both positive and negative) changes in stunting are well-understood. Evidence around nutrition outcomes is necessary for creating enabling environments to address undernutrition (Gillespie et al., 2013). Understanding stunting changes can thus guide evidence-based nutrition decision making and evaluation of policies and programmes and propel and sustain momentum for scaling up coverage of essential interventions and reducing undernutrition, accelerating progress towards achieving national nutrition targets and the 2025 and 2030 nutrition targets of the World Health Assembly (WHA) and Sustainable Development Goals (SDGs), respectively. Specifically, the WHA endorsed a target of 40% reduction in the number of stunted under-five children by 2025 and the SDGs aim to achieve internationally agreed targets for under-five stunting by 2030. Based on the WHA target, the Nigeria National Policy on Food and Nutrition (MBNP, 2016) includes a target to “*reduce stunting rate among under-five children from 37% in 2013 to 18% by 2025*” (pg. 10), representing a 51% reduction in prevalence.

Several previous studies, for example Adekanmbi et al. (2013), Ajieroh (2009), Akombi et al. (2017), Uthman (2009), have assessed the determinants of stunting in Nigeria, but these studies mostly used a single round of the Nigeria Demographic Health Survey (NDHS) data or focused on changes in stunting through a socioeconomic inequality lens. Two studies (Akombi et al., 2019; Nwosu & Orji, 2018) assessed changes in socioeconomic inequalities of child stunting over time, using 2003, 2008, and 2013 NDHS data. In addition to focusing on socioeconomic inequalities, neither of these two studies included health and maternal nutrition determinants of child stunting. Other authors (Amare et al., 2018) conducted a decomposition analysis to elucidate the difference in stunting for children 6 – 23 months old, between northern Nigeria (North East (NE) and North West (NW) geopolitical zones combined) and the rest of Nigeria, using 2013 NDHS data. In general, the studies reported that stunting was higher among the poor, rural households, and households in northern Nigeria. Low maternal education and nutrition, inadequate use of health services, unimproved drinking water source, poor sanitation, illness, and inadequate child feeding practices were other factors associated with stunting.

Although these studies offer useful explorations into the determinants of stunting in Nigeria, none analyze the factors contributing to changes in stunting prevalence over time, which is the goal of this study. The specific objectives of this study were to:

1. Assess the changes in height-for-age z-scores (HAZ) and stunting from 2003 to 2018
2. Describe changes over time in the immediate, underlying, basic and enabling environment determinants of stunting
3. Determine factors associated with HAZ and stunting and decompose the determinants contributing to observed changes in HAZ and stunting
4. Project potential 2025 stunting levels if predicted determinants of stunting improve

## Materials and methods

This study is part of the Stories of Change in Nutrition study (SoC) in Nigeria conducted by the Transform Nutrition West Africa project (TNWA). The analysis conducted used the methods described in Headey et al. (2015) and Headey and Hoddinott (2015), who proposed regression-decomposition methods to explore potential drivers of stunting change. The basic idea of this approach is to ask how well a given stunting regression model predicts stunting changes over time – for example, across different DHS rounds – based on changes in means for the explanatory variables (the determinants of stunting change) in the regression model.

### Hypothesized drivers of changes in growth outcomes

Since 2001, Nigeria has had a National Policy on Food and Nutrition that describes different approaches to addressing malnutrition in the country. Similarly, many policies, strategies, and plans of action for improving health, agriculture, economic growth, education, environment, social protection, and other nutrition-sensitive issues have been developed. Further, Nigeria became a member of the Scaling Up Nutrition (SUN) movement in 2011, working towards scaling-up existing multisectoral nutrition actions in the country to accelerate progress on reducing undernutrition, and more recently all forms of malnutrition. Informed by existing literature (Amare et al., 2018; Headey & Hoddinott, 2015; Headey et al., 2015), potential determinants of stunting change that were considered in the decomposition analysis in this study included economic growth (and its potential impacts on household wealth); improvements in education; and expansion of nutrition, health, family planning, and water and sanitation interventions.

### Data

The analysis was conducted using 2003, 2008, 2013 and 2018 NDHS data for children 0 to 59 months old (NPC & ICF, 2019; NPC & ICF International, 2014; NPC & Macro, 2004, 2009).^1^ The 2003 NDHS was representative at the national, rural/urban, and geopolitical zone level only, while the 2008, 2013, and 2018 NDHS were representative at these levels as well as at state levels. Households included in the survey were sampled using a complex two-stage cluster randomized sampling design, with survey weights used to ensure representativeness at the national or subnational levels.

The national analysis included a total of 57,507 children with HAZ ± 6 SD from the median, based on the 2006 WHO child growth reference population. The 2003, 2008, and 2013 NDHS collected anthropometric data for all under-five children in all selected households, while the 2018 NDHS collected anthropometric data from under-five children in one-third of selected households. Thus, there were 4,174 children from 365 clusters in 2003 NDHS, 18,419 from 886 clusters in 2008, 23,835 from 896 clusters in 2013 and 11,079 from 1,389 clusters in 2018 NDHS. Online Resource 1 in the supplementary material summarizes the indicators representing the determinants of stunting change considered in the study and defines how the indicators were constructed. Online Resource 1 also includes a description of outcome and control variables. Indicators were generally constructed guided by Headey et al. (2015, 2017) and Headey and Hoddinott (2015). Included determinants encompassed the immediate (dietary intake where possible, and child illness – diarrhoea, fever, or cough); underlying (maternal nutrition, water/sanitation, health care); and basic (assets, education, demographic changes) determinants of nutritional status (Black et al., 2013).

In addition, markers of potential intrasectoral and multisectoral coordination were assessed as indicators of the nutrition enabling environment (Black et al., 2013). Potential intrasectoral coordination was defined as the delivery of several interventions that takes place consecutively and are implemented by various units or actors within the same sector, such as coordinated “continuum of care” health services during the first 1000 days of life (Kerber et al., 2007). Potential multisectoral coordination was defined as the delivery of a number of interventions that takes place concurrently but are implemented by actors from multiple sectors (Garrett & Natalicchio, 2011). The potential coordination indicators were selected based on three eligibility criteria: 1) already included as a stunting determinant in the study (Online Resource 1); 2) measures outcomes targeted in national policies/strategies; and 3) has standards or cut-offs established in literature (for example, there are no cut-offs for “acceptable levels” of asset index, hence, it was not included in assessing potential coordination).

For potential intrasectoral coordination, continuum of care within the health sector was assessed (Kerber et al., 2007), as this was the only sector for which multiple indicators along a continuum were available. Specifically, a dummy indicator (0/1) was generated with dummy = 1 if all of three conditions were met: mother had at least 4 ANC visits; child was born in a medical facility; and child had received all age-appropriate basic vaccinations. For potential multisectoral coordination, a dummy indicator (0/1) was generated with dummy = 1 if all of six conditions were met: 3 potential health intrasectoral coordination indicators; child household used piped, borehole, tube well or covered well drinking water; household did not engage in open defecation; and mother or partner had at least primary education (6 years). Further, a discrete variable was generated with values ranging from 0 to 6, based on the number of the six conditions that were met for a child. These are only indicators of potential coordination – it is possible that favorable outcomes across or within sectors are not driven by coordinated policies but by other factors (e.g., household wealth and greater demand).

### Data analyses

The analysis adjusted for the complex survey design of the DHS, using the *svyset* and related commands in Stata 15.0 that consider sampling units, strata, and sample weights; and the probability of decision error, α, was set at 0.05. Descriptive statistics included trends in means and prevalence, as well as non-parametric plots of HAZ by child age, following Headey and Hoddinott (2015). Univariate regression models and chi-square tests were used to assess the significance of change in means/prevalence from 2003 to 2018. Given the differences in malnutrition prevalence across geopolitical zones and states (NPC & ICF, 2019; NPC & ICF International, 2014; NPC & Macro, 2009), as well as state autonomy in nutrition decision-making, analysis was done at national, geopolitical zone, and state levels. For state level analysis, two states were selected – one state that appeared to be performing relatively well for stunting and one state that was not performing well. This selection was made in order to facilitate an understanding of factors enabling positive change or limiting change in stunting at state level as part of the broader SoC in Nigeria. In addition to analysis of past changes in stunting, potential changes that could result from scaling up efforts to improve nutrition were projected, following methods used by other authors (Headey et al., 2019). Such projections facilitate an understanding of changes that will be required in various interventions to achieve targets for stunting reduction (Headey et al., 2019).

#### National level

For regression analyses, child growth outcomes – child HAZ, stunting (HAZ < –2 SD), and severe stunting (HAZ < –3 SD) – were the dependent variables of interest. Linear regression (ordinary least square, OLS) models were used where child HAZ was the outcome, while linear probability models (LPM) were used for stunting. Apart from models that directly included all the driver indicators, additional models were run where the discrete (0 to 6) multisectoral coordination variable replaced the following indicators: piped water, borehole, open defecation, antenatal care, health facility delivery, vaccination, maternal education, and paternal education.

Data from the 2003, 2008, 2013 and 2018 NDHS surveys were pooled and modelled using Eq. (1), where:

- **N** is the nutrition status indicator (HAZ or stunting) for a child *i* at time *t*
- **X** is a vector of time-varying indicators (determinants of nutritional change)
- ***β*** is a vector of coefficients which are parameters of interest to be estimated
- ***μ*** is a vector of control variables including maternal height, maternal and child age dummies, and location (rural/urban) fixed effects
- **T** is a set of time dummies for each round
- ***ε*** is the error term
  1 $${N}_{i,t}={\varvec{\beta}}{{\varvec{X}}}_{i,t}+{{\varvec{\mu}}}_{{\varvec{i}}}+{\varvec{T}}+{\varepsilon }_{i,t}$$

Equation (1) requires that non-linear relationships are identified and addressed and that there is little collinearity among the variables in the model. To ascertain that the assumption of linearity was met, non-parametric graphical techniques were used to examine time-varying continuous variables (Online Resource 2), while flexible specifications (for instance monthly dummy variables for child age) were used for time-invariant variables. For the collinearity assumption, pairwise correlations were done between all continuous variables to ensure that no variables were highly correlated. Variance inflation factors (VIFs) were also assessed to ensure that no variable had a high VIF (Online Resource 3). In the full national sample, nearly all the continuous variables included in the model had approximately linear relationships with the HAZ outcome. Cluster level open defecation showed some signs of non-linearity but was kept as linear in the main set of results. As a sensitivity test, additional analysis (Online Resource 4) breaks the open defecation variable into five quintiles (based on the curves of its non-parametric graph) and checks the effects on regression coefficients. Amare et al. (2018) suggested that there may be a threshold effect for some determinants. Hence, the non-parametric graphs (Online Resource 2) were also examined to determine the possibility of thresholds at which the predicted –2 HAZ line crosses the x-axis in each graph and identify the corresponding value of the driver indicator at the threshold. The –2 HAZ line is the threshold below which a child is said to be stunted. Thus, the point at which this line crosses the x-axis may indicate the minimum levels of a determinant that is necessary to avoid stunting.

The estimated parameters from Eq. (1) were used to conduct a decomposition analysis of significant changes in growth outcomes, with a simple decomposition of means. The predicted change in growth outcome due to any variable X was estimated as the change in the mean of X multiplied by its regression coefficient from Eq. (1). The model used only regression coefficients that were significant at < 5%. This model assumes that the ***β*** coefficients remain constant across survey years and that the error term has a mean of zero. The decomposition equation is therefore:

2 $${\Delta \overline{N} }_{T}={\varvec{\beta}}\left({\overline{{\varvec{X}}} }_{t=T}-{\overline{{\varvec{X}}} }_{t=1}\right)$$

where $\overline{\mathbf{X} }$ refer to sample means derived from survey weights.

To check that the assumption of coefficient stability was valid, Chow tests were conducted for significant differences between the coefficients of each X variable across the four survey rounds, as well as between the coefficients of the pooled model and coefficients in each survey round. The robustness of the LPM for stunting and severe stunting was assessed by comparing the β-coefficients from the LPM to marginal effects estimated from logistic regression models of the outcomes. To assess the potential inclusion of endogenous variables, models that excluded health and fertility variables were estimated, as has been done by other authors (Headey et al., 2015), because demand for health services and contraception is typically considerably increased by wealth and education. Clustered robust standard errors were used to estimate significance levels in all models. Also, to prevent regression coefficients from being weighted by the data from one year, due to the large sample size differences across the survey years, new weights were constructed using survey population weights and the inverse of the sample size proportion from each year. The newly constructed weights were used in regression models while original population weights were used for descriptive statistics for each NDHS round.

The full national sample analysis was replicated by type of place of residence (rural/urban) and age group (< 6 months, 6–23 months, and 24–59 months). Previous studies reported significant rural/urban differences in stunting changes (Smith et al., 2005) as well as age group differences (Alderman & Headey, 2018); hence the choice of subsamples for analysis. In addition, the quality of Nigeria DHS data has been shown to be quite poor (Fayehun et al., 2020; Larsen et al., 2019; Nwogu, 2006; Perumal et al., 2020), with some, but inadequate quality improvements in successive surveys (Perumal et al., 2020). The poor data quality is more pronounced for rural areas and northern geopolitical zones, with the NE and NW zones having the poorest data quality (Fayehun et al., 2020). To minimize the potential for systematically biased results due to poor data quality, this study used national coefficients for decomposing any changes in HAZ and stunting in location-specific samples such as rural/urban analysis (i.e., rural/urban (and other location) child anthropometric and determinants data were used, but the β-coefficients from the pooled sample were used to decompose the change over time).

#### Geopolitical zone level

The pooled model had 9,752 children in the North Central (NC), 11,969 children in the NE, 15,306 children in the NW, 5,794 children in the South East (SE), 6,778 children in the South South (SS), and 7,908 children in the South West (SW). HAZ and stunting regression models for each zone were analyzed to provide indication of coefficients that likely differed across zones. Zonal decomposition of changes in HAZ and stunting was conducted in zones where significant changes occurred from 2003 to 2018. Again, coefficients from the pooled national model were used for such analysis, to minimize data quality bias and avoid any unstable zonal coefficients. The use of national coefficients also permits an examination of how well the national model predicted zonal trends, or how well/badly the model worked in different zones. However, such decomposition implies an assumption of uniform coefficients across zones.

#### State level

State level analysis was conducted from 2008 to 2018 because 2008 was the first year for which the NDHS collected representative data at state level. At the time of study commencement (January 2019), the most current state-level representative data was from the 2016/17 Nigeria Multiple Indicator Cluster Survey (MICS). There are historical and fundamental differences among geopolitical zones in Nigeria (Archibong, 2018; Eze et al., 2014). Jigawa State in the NW zone was selected as a case study for a state that was relatively not well-performing, because prevalence of stunting had increased between 2008 (53%) and 2016 (66%). Jigawa also had the highest prevalence of stunting in 2016 (NBS & UNICEF, 2017). States in southern Nigeria had the lowest prevalence of stunting and apparent reductions in prevalence from 2008 to 2016. However, we chose a relatively well-performing state from the same geopolitical zone as Jigawa – Kaduna state – in order to reduce potential confounding due to zonal factors. Kaduna was selected as the well-performing state because it had the lowest prevalence of stunting in the NW in 2016, as well as the largest apparent reductions in stunting prevalence in the zone from 2008 (52%) to 2016 (47%). Pooled data from 2008 to 2018 had 4,058 and 3,324 children in Jigawa and Kaduna, respectively. Changes over time in the prevalence of stunting determinants that were significant in the national level decomposition analysis were assessed for each state.

#### Projection analysis

Potential stunting changes from 2018 to 2025 due to intervention efforts were projected using Eq. (2). Specifically, two scenarios were modelled. For scenario one, it was assumed that the annual rate of change for each determinant from 2018 to 2025 would be the same as the average annual rate of change from 2003 to 2018. Scenario two set the 2025 mean of each determinant in the decomposition model to the mean in the best performing geopolitical zone (for that determinant) in 2018.

## Results

Overall, progress in stunting reduction in Nigeria has been slow but also highly uneven. The regression models shed light on factors associated with stunting reduction. Model projections to 2025 suggest that stronger multisectoral efforts with greater coverage could deliver much faster progress against stunting.

### National level

#### Descriptive statistics:

**Fig. 2:**
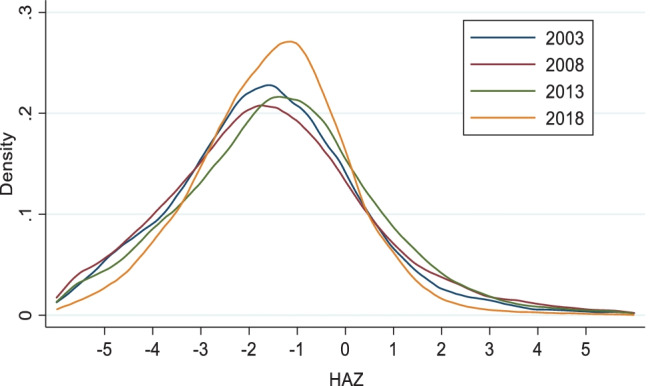
Distribution of Length/Height for Age Z-Scores from 2003 to 2018 in Children 0–59 Months Old

**Fig. 3:**
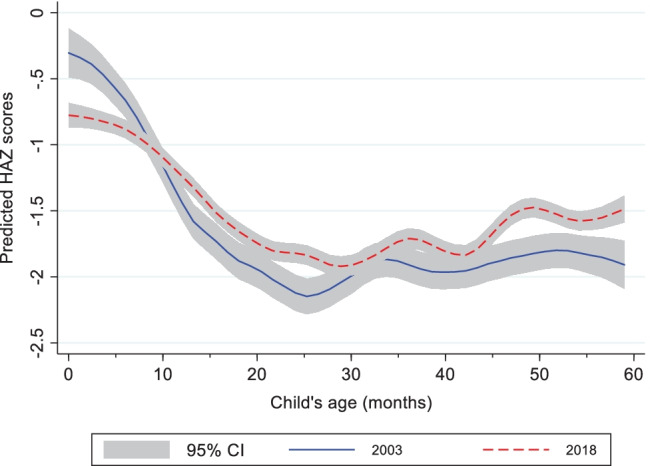
HAZ by child age, 2003 and 2018

There was a non-significant increase of 0.10 in mean HAZ from 2003 to 2018 in the full sample, while both stunting and severe stunting significantly reduced by 6 percentage points over the period (Table 1). Changes in HAZ and stunting were not consistent across the years (Online Resource 5) and the reduction in stunting mostly occurred in the 2008 to 2013 period. The limited changes in HAZ from 2003 to 2018 can be observed in the kernel density estimation of the HAZ distribution (Fig. 2). Although the 2018 curve shifted to the right, the right-side tail constricted. There is therefore a greater clustering of children around the mean than in earlier survey years. When HAZ was observed by child age in months (Fig. 3), predicted HAZ were significantly lower at birth in 2018 than in 2003 (confidence intervals do not overlap). However, the slope of post-natal growth faltering is less steep in 2018 than in 2003 in the first ± 24 months.

**Table 1:** Nutrition Outcomes and Determinants of Nutrition for Children 0 to 59 Months Old in 2003 and 2018

**Characteristics**	**2003**	**2018**	**% ∆**
***Outcomes***			
Mean HAZ	-1.62 (1.90)	-1.52 (1.58)	*6.21*
% Stunting (HAZ < – 2)	42.56	36.54	-14.13***
% Severe Stunting (HAZ < – 3)	22.79	16.95	-25.63***
***Maternal Nutrition***			
% Underweight (BMI < 18.5 kg/m^2^)	12.32	9.39	-23.72***
Average height (cm)	158.39 (6.08)	158.43 (5.85)	*0.02*
***Health and Health Seeking***			
% ANC at Least Four Visits	51.46	61.79	20.07***
% Delivery in Health Facility	36.61	45.58	24.49***
% Children Fully Vaccinated at Appropriate Age	11.68	23.99	105.34***
% Child Illness in 2 Weeks Preceding Survey	46.81	36.58	-21.86***
***Water and Sanitation***			
% Piped Drinking Water Source	15.53	10.81	-30.37***
% Borehole/Covered Well Drinking Water Source	21.59	48.46	124.42***
% Households with Open Defecation	24.53	23.45	*-4.42*
***Wealth and Education***			
Mean Asset Index	3.53 (2.88)	4.08 (2.77)	15.73***
Mean Maternal Education (years)	4.29 (4.82)	6.28 (5.72)	46.39***
Mean Paternal Education (years)	5.85 (5.66)	7.41 (6.07)	26.75***
***Demography***			
Mean Number of Children per Woman	4.38 (2.67)	4.20 (2.51)	-4.13**
% Birth Interval Less than 18 Months	6.33	6.03	*-4.70*
***Enabling Environment***			
% Received 0 Health Sector Continuum of Care Actions	48.78	35.94	-26.32***
% Received 3 Health Sector Continuum of Care Actions	6.33	12.17	92.33***
% Received 0 of 6 Multisectoral Determinants	6.90	3.94	-42.94***
% Received 6 of 6 Multisectoral Determinants	3.28	6.44	96.61***

Numbers in parentheses are standard deviations

*** and ** indicate significance at 1% and 5% levels, respectively

Determinants of nutritional status generally improved from 2003 to 2018. Despite the improvements, levels of determinants were nonetheless low in 2018. For instance, in 2018, 62% of women received antenatal care at least four times, 46% of children were born in a medical facility, and 24% of children had received all age-appropriate basic vaccinations. Further, just 12% of children received all three of these interventions, and 36% of children did not receive any of the three interventions. Around 40% of households did not have adequate drinking water sources, and 23% still practiced open defecation. Mean asset index was 4 out of 10. Average number of years of education completed was 6 years for mothers and 7 years for fathers. In 2018, just 6% of children received all the selected six interventions that comprise the potential multisectoral coordination indicator; compared to 3% of children who had received the six interventions in 2003 (Table 1).

#### Factors explaining growth outcomes in Nigeria:

Determinants included in the models were generally associated with the outcomes in the expected directions. Asset index, maternal and paternal education, maternal height, having ≥ 4 ANC visits, and health facility delivery were positively associated (p < 0.05) with child growth; while increasing fertility, low birth intervals, low maternal BMI, open defecation, and child illness were negatively associated. Being a boy child and living in a rural residence were also negatively associated with growth (Table 2). When the number of multisectoral indicators each child received replaced its constituent variables in regression models, it was observed that improved child growth was associated with increasing numbers of indicators registered (Online Resource 6). Notably, receiving ≥ 4 of any combination of the selected 6 multisectoral determinants predicted significantly improved growth outcomes, compared to receiving zero of the determinants.

**Table 2:** HAZ and stunting regressions pooled across 2003, 2008, 2013 and 2018 NDHS for full national sample

	Full National Sample, HAZ OLS	Full National Sample, Stunting LPM	Full National Sample, Severe Stunting LPM
Low maternal BMI	-0.261***	0.062***	0.038***
	0.039	0.010	0.009
Maternal height (cm)	0.037***	-0.008***	-0.005***
	0.002	0.000	0.000
≥ 4 ANC visits	0.096	0.007	-0.063***
	0.069	0.014	0.012
Delivery in health facility	0.132***	-0.038***	-0.014**
	0.029	0.007	0.006
Complete age-appropriate vaccinations	0.02	-0.011*	-0.008
	0.029	0.007	0.005
Child illness	-0.157***	0.026***	0.033***
	0.024	0.006	0.005
Piped water	-0.02	0.002	0.000
	0.042	0.010	0.009
Borehole/covered well water	-0.024	-0.005	-0.002
	0.026	0.006	0.005
Open defecation	-0.120**	0.014	0.01
	0.049	0.009	0.008
Asset index (1 – 10)	0.051***	-0.014***	-0.006***
	0.007	0.002	0.001
Maternal education (years)	0.013***	-0.004***	-0.003***
	0.003	0.001	0.001
Paternal education (years)	0.005*	-0.002**	-0.002***
	0.003	0.001	0.001
Number of children per woman	-0.017**	0.003*	0.000
	0.007	0.002	0.002
Low birth interval	-0.148***	0.041***	0.039***
	0.045	0.012	0.011
Male child	-0.201***	0.052***	0.035***
	0.022	0.005	0.005
Rural residence	-0.074**	0.023***	0.012**
	0.035	0.007	0.006
Year 2008	0.170***	-0.014	0.007
	0.054	0.011	0.01
Year 2013	0.354***	-0.069***	-0.022*
	0.069	0.015	0.013
Year 2018	0.162*	-0.053***	-0.049***
	0.084	0.020	0.017
R-squared	0.209	0.177	0.139
N	57507	57507	57507

Clustered, robust standard errors are below point estimates

The regressions included several time-invariant controls, including zonal fixed effects, dummy variables for practice of Christianity and Islam, month-specific child age dummy variables, and dummy variables for various categories of maternal age and maternal cohort

*OLS* ordinary least square model, *LPM* linear probability model

***, ** and * indicate significance at 1%, 5%, and 10% levels, respectively

#### Decomposition of growth trends:

**Fig. 4:**
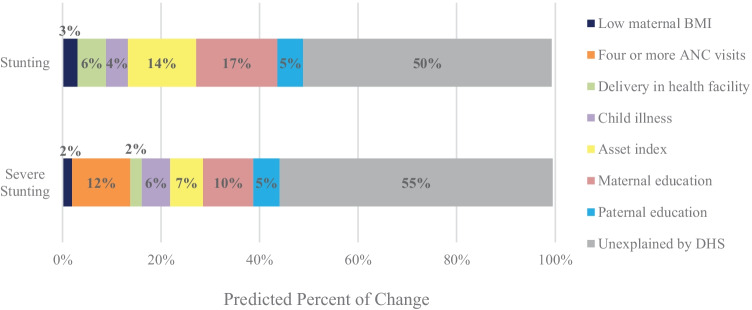
Predicted sources of changes from 2003 to 2018 in stunting and severe stunting prevalence in Nigeria

Figure 4 and Online Resource 7 present the results of decomposing changes in growth outcomes nationally. Increases in parental (maternal and paternal) education was predicted to have contributed the most to the decline in stunting from 2003 to 2018; followed by the increase in assets, improvements in health (increased health facility delivery and reduced child illnesses) and improved maternal nutrition (reductions in low BMI and increased maternal height). In addition to these determinants, improvements in the proportion of women that had ≥ 4 ANC visits also contributed to the predicted reductions in severe stunting. The predicted models did not explain all of the observed change in stunting and severe stunting from 2003 to 2018. Specifically, half (50%) of change in stunting (–3 percentage points (pp) out of –6 actual pp) and 45% of change in severe stunting (–2.6 pp out of –5.8 actual pp) was predicted by the models (Fig. 4 and Online Resource 7). In addition, the acceleration in stunting reduction observed between 2008 and 2013 appeared to be influenced by improvements in maternal height during this period (Online Resource 7).

There were significant differences in growth outcomes and determinants between rural and urban areas (Online Resource 8). Summarily, rural areas did not see a statistically significant decline in stunting or severe stunting from 2003 to 2018, while urban areas witnessed significant declines in both outcomes (p < 0.05). Although rural areas had greater 2003–2018 improvements in several determinants (including vaccination, ≥ 4 ANC visits, and borehole drinking water source), the 2018 coverage of determinants in rural areas was still below urban coverage (Online Resource 8A). Factors associated with HAZ, stunting, and severe stunting, were similar for rural and urban areas (Online Resource 8B), as with the full sample, with a few notable exceptions. Child vaccinations, open defecation, and low birth intervals were significant in urban but not rural areas; while low maternal BMI, child illnesses, health facility delivery, and paternal education were significant in rural but not urban areas.

There were likewise important age group differences in the changes in growth outcomes and determinants (Online Resource 9). Stunting reductions were observed among children 6 to 59 months old but there was no change among children 0 to 6 months old (Fig. 3, Online Resource 9A). Among nutrition determinants, nutritional status improved among mothers of children 6 to 59 months old, but not among mothers of children 0 to 6 months old. Exclusive breastfeeding increased among children 0 to 6 months old, but complementary feeding deteriorated among children 6 to 23 months old. The prevalence of younger children (0 to 23 months old) that received the 3 selected health interventions along a continuum of care meaningfully increased, whereas there was no change among children 24 to 59 months old (Online Resource 9A). Maternal height and ≥ 4 ANC visits were the only determinants consistently associated with growth outcomes in all 3 age groups. The models identified only a few factors significant for growth outcomes among children < 6 months old while many of the determinants were significant for children 24 to 59 months old (Online Resource 9B and C).

### Geopolitical zone level

#### Descriptive statistics:

**Fig. 5:**
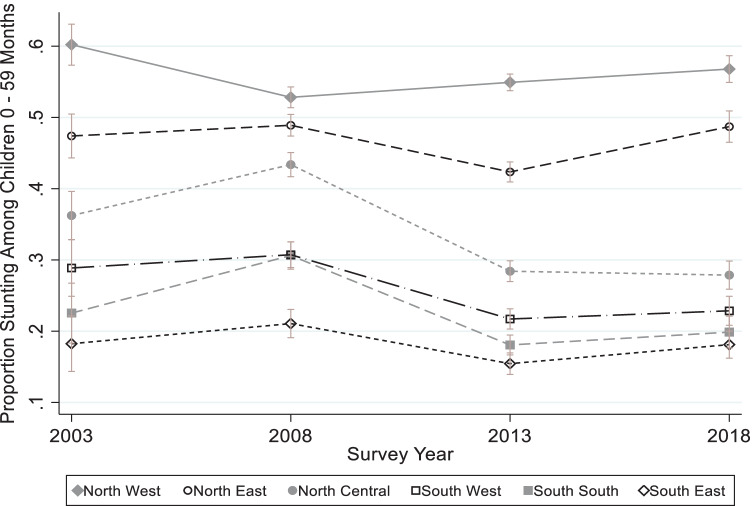
Changes in Stunting Proportion among Children 0–59 Months Old from 2003 to 2018 by Geopolitical zone

At the geo-political zone level, mean HAZ did not change from 2003 to 2018 (p > 0.05) in all zones. The lower predicted HAZ at birth in 2018 that was observed in the full sample (Fig. 3), was observed in three geo-political zones (Online Resource 2) – NC, NE, and SS. Just two geo-political zones experienced statistical declines in stunting from 2003 to 2018, the NC and SW. Similarly, severe stunting declined in the NC and NW only (Online Resource 10). Reductions in stunting mostly occurred in the 2008 to 2013 period, except in the NW where stunting significantly increased (p = 0.022) from 2008 to 2018 (Fig. 5 and Online Resource 5). In the most recent period, i.e., from 2013 to 2018, HAZ significantly (p < 0.05) deteriorated in all zones except in the NW zone where it did not change. Also, stunting increased in the NE from 2013 to 2018 (p = 0.004). Overall, the SE consistently had the lowest prevalence of stunting, followed by the SS zone, while the NW and then the NE consistently had the highest prevalence of stunting (Online Resource 10A).

Like nutrition outcomes, there were variations in the magnitude and direction of change in determinants of nutrition across the zones (Online Resource 10A). Determinants did not consistently change in the same direction across the zones. For instance, whereas the prevalence of completion of age-appropriate vaccination increased about 15 times in the NW, it decreased by 24% in the SW. Also, while there was 46% reduction in open defecation in the NW, there was a 23% increase in the NC. There was no zone where all determinants of stunting improved. The NW and NE zones have the greatest potential to benefit from any interventions to improve nutrition because, in both 2003 and 2018, they had the highest prevalence of determinants of child growth retardation. Because of the magnitude of need in 2003, even large improvements from 2003 to 2018 in these zones did not necessarily mean good coverage in 2018. For example, the NW prevalence of complete age-appropriate vaccinations was 0.8% in 2003 and 13% in 2018. This 15 times vaccination improvement in the NW was the highest improvement across all zones. Yet, the NW still had the lowest coverage in 2018.

#### Factors explaining growth outcomes:

Non-parametric graphs for the NW, but not any other zones (Online Resource 2), suggest that some determinants may have thresholds below which stunting does not meaningfully decline. Specifically, there appeared to be thresholds of around 4 for asset index, 8 years for maternal education, 10 years for paternal education, 55% of households in a village receiving ≥ 4 ANC visits, and maternal height ≥ 160 cm. These numbers represent the point at which the –2 predicted HAZ line crossed the x-axis.

In the regression models, the only variables that were consistently associated with growth outcomes in all zones were maternal height and BMI, and asset index, and the predicted impact of these variables was quite considerable. In all zones, male children also consistently had poorer growth outcomes than female children. All other variables were significant in some but not all zones, with different magnitudes of coefficient even where significant and with even differing directions of coefficient in a few instances (Online Resource 10B-D). The ability of decomposition models to adequately predict changes in stunting was reduced because a number of the determinants that predicted stunting change deteriorated in several zones (Online Resource 10E).

### State (Kaduna and Jigawa) level

Child HAZ significantly deteriorated (p < 0.001) and stunting statistically increased (p = 0.005) in Jigawa from 2008 to 2018, whereas there was no change in Kaduna for both HAZ and stunting (Online Resource 11). Further, while the prevalence of severe stunting did not change in Jigawa (p = 0.641) from 2008 to 2018, there was a significant decrease in Kaduna during this time period (p = 0.017). Although there were improvements in several nutrition determinants, including vaccinations and reductions in open defecation, only a few of the predicted sources of stunting decline improved in Jigawa from 2008 to 2018 (Fig. 4 and Online Resource 7). Specifically, of nine indicators that predicted stunting and/or severe stunting, ANC visits, health facility delivery, and paternal education were the only indicators that improved. The other indicators did not improve or deteriorated (child illness increased by more than 100%). In Kaduna, none of the nine indicators improved from 2008 to 2018. Rather, child illness and low birth intervals increased. Hence, our model did not work well in explaining the differences in stunting changes between Kaduna and Jigawa.

### Potential determinants of future stunting reduction in Nigeria (2018–2025)

**Fig. 6:**
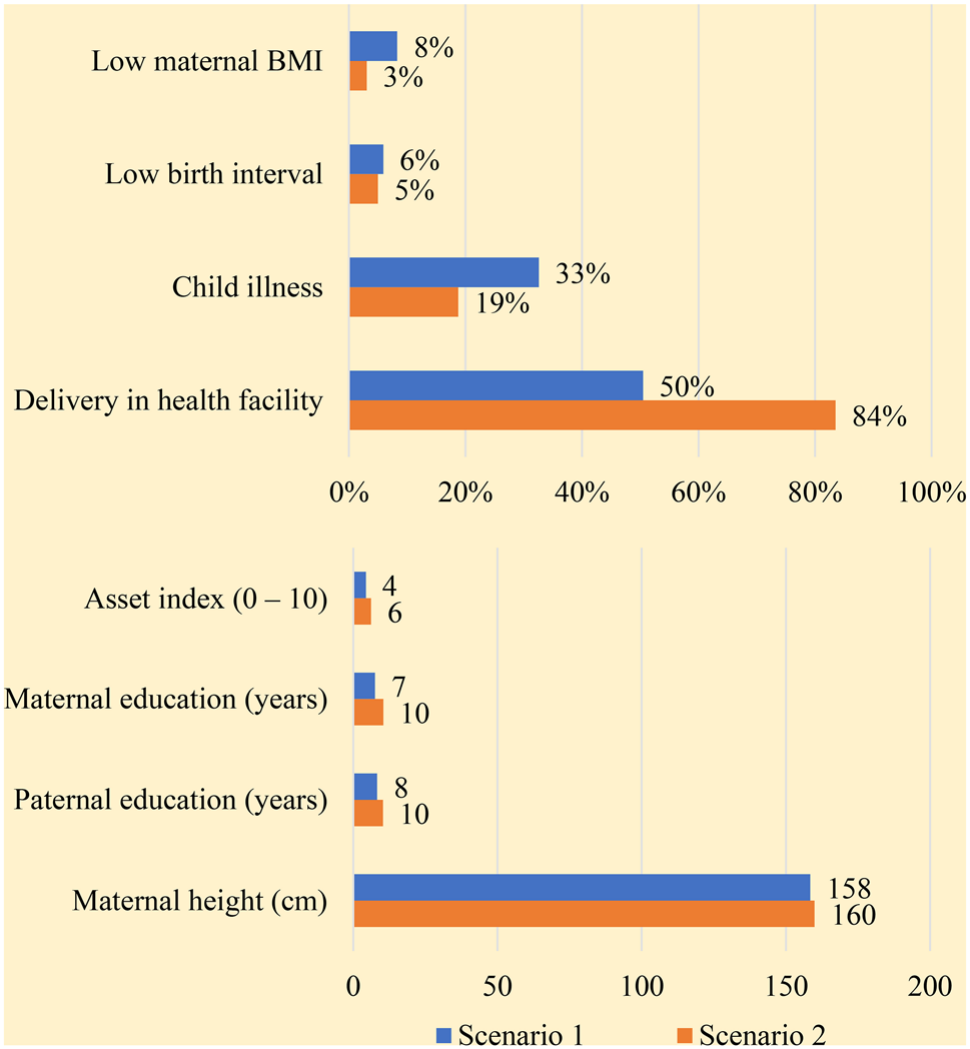
2025 levels of determinants in two scenarios – scenario 1 and 2

**Fig. 7:**
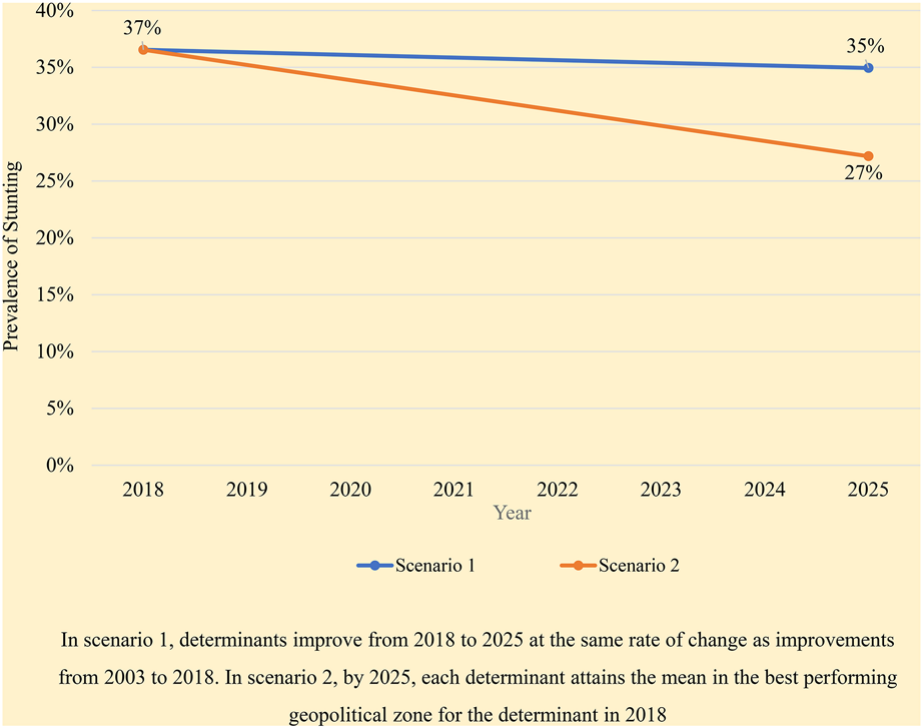
Projected prevalence of stunting in scenario 1 and scenario 2 improvements in determinants

When the coefficients from the decomposition analysis (Online Resource 7) were used to project two scenarios of stunting levels in 2025, the gaps between current levels of determinants and the levels necessary for meaningful change were amplified. In the first scenario in which 2018–2025 changes in stunting determinants occur at the same annual rate as 2003–2018 changes, the predicted prevalence of stunting in 2025 will be 34.9%, two percentage points lower than the 2018 prevalence. If, however, prevalence of determinants is considerably improved so that 2025 national means are equivalent to means in the best performing geopolitical zone in 2018 (scenario 2), the predicted prevalence of stunting in 2025 will be nine percentage points lower at 27.2% (Figs. 6 and 7; Online Resource 12).

### Model specification

There were some unstable coefficients among the 2008, 2013 and 2018 rounds and between the 2008 and 2013 rounds and the pooled model (Online Resource 13). Coefficients most affected were maternal height, number of children, complete vaccinations, and child illness. Between the 2003 round and the other three rounds, and between the 2003 round and the pooled model, there were no unstable coefficients. Also, between the 2018 round and the pooled model, open defecation at the cluster level was the only unstable coefficient. In 2018, there was no relationship between open defecation and HAZ, while the pooled model showed a significant negative relationship. Nevertheless, there was only a 1.1 percentage points change in the prevalence of open defecation between 2003 and 2018, indicating that the influence of this coefficient on decomposition results will be minimal. Since the decomposition analysis used 2003 and 2018 data and there were basically no signs of coefficient changes between these rounds or with the pooled model, the linear decomposition method was considered suitable.

Regarding the potential inclusion of endogenous variables, the models excluding health and fertility variables found that the coefficients for assets increased by 29% and 67% for stunting and severe stunting, respectively; while coefficients for maternal education increased by 50% and 33%, respectively (Online Resource 14A). The larger coefficients for asset index and maternal education indicate that a larger share of the change in stunting and severe stunting may be attributable to improvements in these two determinants. Nevertheless, the decomposition results including asset index and parental education alone explain less of the change in stunting and severe stunting (42% and 28%, respectively) than the full model (Online Resource 14B). The results therefore support an important role for health and fertility determinants in reducing stunting and severe stunting. The logit models produced generally similar patterns of significant variables compared to the LPM model (Online Resource 15A). Inferences about the contributions of the various indicators to predicted nutritional change were likewise similar (Online Resource 15B).

## Discussion

This study aimed to assess child growth outcomes in Nigeria, associations between these outcomes and nutrition determinants, likely contributions of determinants to changes in growth outcomes from 2003 to 2018, and projected stunting reductions if determinants are improved by 2025.

### Contributions to knowledge about stunting drivers in Nigeria

The 2018 prevalence of stunting in Nigeria (37%) is classified as critical (de Onis et al., 2019). The findings of our study provide insights into the limited progress in reducing stunting in Nigeria. Specifically, five key conclusions are supported. Our first key finding is that determinants of stunting, especially wealth and maternal education, that facilitate the demand for nutrition-promoting services, are crucial in Nigeria. Secondly, supply-side determinants, including access to health care, family planning, and infrastructure, are also important. Both findings have been reported in other studies of stunting determinants in Nigeria, such as Adekanmbi et al. (2013), Ajieroh (2009), Akombi et al. (2017), and Amare et al. (2018). The added value of our study was to estimate the past and potential future contributions of the various determinants to stunting reductions. Nationally, we found that education predicted the most change in stunting reduction (22%), followed by wealth (14%), health (10%), maternal nutrition (3.5%), and fertility (0.2%). Our findings are consistent with previous studies. A review of child growth decomposition analyses in 11 countries and 4 states of India (Heidkamp et al., 2021) found that wealth, healthcare, and parental education were top drivers of stunting reduction. The findings emphasize the need for multisector interventions to reduce stunting.

Our third key finding is that significant determinants of stunting reduction appeared to vary within country from rural to urban areas, from one geopolitical zone to another, and among different age groups. For instance, we found that open defecation had greater significance in urban areas (consistent with a global study using DHS data – (Hathi et al., 2017), and vaccinations were consistently significant in the SE zone for all growth outcomes. Some authors have highlighted within country differences in child growth outcomes, e.g. (Vaivada et al., 2020). However, differential effects of various determinants across subpopulations appear to have been much less studied beyond rural/urban differences, e.g., Smith et al. (2005) and Hathi et al. (2017); and age group differences e.g., Alderman and Headey (2018) and Svefors et al. (2019). One study, Amare et al. (2018), studied differences in effects of determinants on stunting outcomes between Nigeria NE and NW zones, and the other four zones of the country, among children 6 to 23 months old. Similar to our study, the authors reported variations in the effects of determinants on stunting outcomes. Although the observed differential effects might be due to systematic measurement error arising from poor quality of Nigeria DHS data (Fayehun et al., 2020; Larsen et al., 2019; Perumal et al., 2020), the finding suggests a need to consider the possibility of differential effects when selecting and targeting interventions.

The significantly lower HAZ among children < 6 months old in 2018 compared to 2003 is worrisome as it indicates that postnatal HAZ in Nigeria may be deteriorating, presenting potential risks for stunting increases, and potentially indicating deteriorating maternal nutrition, at least during pregnancy. Yet, the less steep gradient in HAZ reduction between 0 and 6 months (Fig. 3) suggests improved rates of early initiation of breastfeeding and exclusive breastfeeding (Argaw et al., 2019; Hanley-Cook et al., 2020). NDHS data does show that exclusive breastfeeding significantly increased from 20 to 31% from 2003 to 2018 (Online Resource 9A). Hence, the lower HAZ among children 0–6 months appears to be driven predominantly by lower HAZ at birth. This indication, in parallel with potentially lower maternal height over time among this age group (Online Resource 9A), suggests poor maternal nutrition and health and an intergenerational cycle of malnutrition in Nigeria (Accrombessi et al., 2018; Black et al., 2008).

Our fourth key finding is that improvements in all stunting determinants were limited and varied across subpopulations. Determinants that improved nationally did so only among some subpopulations, while other determinants did not change or deteriorated. Our decomposition analysis thus deductively explains that the lack of stunting decline from 2003 to 2018 among different subpopulations in Nigeria is due to limited improvements in the determinants that predicted stunting change. For example, in the NW zone where stunting is highest, only ANC visits improved among predicted determinants of stunting and/or severe stunting change. Decomposition of HAZ and/or stunting in other countries (Bhutta et al., 2020; Buisman et al., 2019; Headey et al., 2017, 2019; Kohli et al., 2020) found that countries where stunting meaningfully declined experienced considerable improvements in multiple nutrition determinants. Other authors (Husseini et al., 2018) signal that certain thresholds of socioeconomic and environmental conditions may be necessary before stunting can meaningfully reduce. For example, at least 9–10 years of maternal education may be required to achieve stunting declines (Alderman & Headey, 2017; Makoka & Masibo, 2015). Our study showed that maternal education among higher stunting burden subpopulations was well-below this threshold in 2018, even where there were significant improvements from 2003. Also, predicted HAZ from univariate, nonparametric graphs in our study suggest potential thresholds in more indicators than maternal education, including asset index, paternal education, ANC, and maternal height. In higher stunting burden subpopulations, the 2018 levels of these indicators were generally below apparent thresholds.

Several (48) multisectoral policies and strategies, aimed at improving service delivery and prevalence of nutrition-relevant development outcomes, were ratified and active in Nigeria between 2003 and 2018 (Adeyemi et al. Forthcoming). Nutrition-specific policies and strategies increased, as did the inclusion of nutrition objectives and activities in agriculture, economic, education, health, water/sanitation/hygiene (WASH), and social protection policies and strategies. Collectively, the policies and strategies seek to substantially scale up food security; reduce poverty; ensure completion of basic education (6 years primary and 3 years secondary); increase access to basic health services (prioritizing reproductive, maternal, neonatal, child, and adolescent health services); achieve open defecation free communities; improve access to safe water, improve sanitation, and proper handwashing facilities; and increase social safety nets (Adeyemi et al. Forthcoming). The limited progress in nutrition determinants that we found suggests that policy/strategy implementation has been slow, insufficient, and/or ineffective. One challenge for improving determinants is population growth. Nigeria's population increased by more than 60 million people between 2003 and 2018 (World Bank, 2019). Thus, although prevalence of determinants did not improve, actual numbers of people served increased. In fact, growth in improved sanitation was so much slower than population growth that numbers of people lacking improved sanitation increased by 70 million between 1990 and 2015 (FMWR, 2016). It would be important to assess how policy implementation changed in Nigeria over the 2003 to 2018 period and understand reasons why improvements in nutrition determinants are limited.

The fifth key finding is that just a small percent of children was concurrently benefitting from improvements in multisectoral stunting determinants. Stunting occurs from interactions among inadequate food, health, and care (Black et al., 2008, 2013). Yet, the implications of this fact for stunting reduction efforts are often overlooked. Specifically, stunting reduction requires that multisector nutrition determinants are simultaneously addressed (Humphrey et al., 2015; Husseini et al., 2018; Remans et al., 2011). Our study highlights that the majority of children in Nigeria, including non-stunted children, are neither receiving the full range of health sector determinants nor multisector determinants. While our study has focused on stunting, linear growth retardation goes beyond stunting and is a much larger problem. Thus, increasing coverage of multisector determinants for all children, regardless of their stunting status, should be a focus of development efforts (de Onis & Branca, 2016; Leroy & Frongillo, 2019; Perumal et al., 2018). Though implementing integrated programmes that deliver multisectoral interventions is one way to increase such coverage, concurrently targeting the same subpopulations with various sectoral interventions (co-location) can also achieve success (Heidkamp et al., 2021; Levinson et al., 2013).

### Implications of findings for future efforts to address stunting

Nigeria’s National Policy on Food and Nutrition (MBNP, 2016) aims to reduce stunting among children under five to 18% by 2025. Our analysis indicates that eliminating geopolitical disparities in 9 indicators by 2025 has the potential to move Nigeria substantially towards this goal. While it is encouraging that some geopolitical zones have reached target intervention levels, gaps between 2018 and desired levels are wide for high stunting burden zones. Nigeria would have to, within 7 years, scale up interventions far beyond progress achieved in the 15 years from 2003 to 2018, and vis-à-vis population growth. Scale-up will also have to happen concurrently across several sectors in each location. Of the 9 determinants identified, the determinants that must be scaled-up or maintained across zones and in states within zones vary (based on current prevalence of these determinants), indicating necessity for nuanced intervention delivery approaches. Although state-specific challenges to scale-up and convergence of intervention delivery exist, stakeholders interviewed as part of Nigeria Stories of Change study proffered several recommended actions. Moreover, the National Committee on Food and Nutrition, through oversight provided by the Vice President of Nigeria, recently committed to a common results framework for nutrition (Adeyemi et al. Forthcoming). This commitment offers opportunities for strategic targeting and scale-up of interventions, if followed through with effective implementation.

### Study limitations and research gaps

Strengths of our study included the analysis of multiple NDHS rounds covering a period of significant change in HAZ/stunting with variation in performance across regions. The approach agnostically explores different predictors of stunting reduction and can assess which factors might have retrospectively driven stunting change, and which could drive faster change in the future. The study also elucidates how multisectoral actions are required to achieve faster reductions in stunting in Nigeria.

Still, our study had some limitations. NDHS collects cross-sectional data, so our analysis could not allow for time-lag of effects or temporality. Also, our study predicted 50% and 45% of actual declines in stunting and severe stunting, respectively, indicating that variables not included in our model were responsible for other proportions of stunting decline, or that measurement error or misspecification are significant problems (indeed, concerns about the quality of NDHS data are widespread – Fayehun et al., 2020; Larsen et al., 2019; Perumal et al., 2020). Determinants of stunting that were not included in our study due to data limitations include child dietary intakes, maternal dietary intakes and nutrition indicators such as micronutrient status and gestational weight gain (Accrombessi et al., 2018; Black et al., 2008, 2013), birth weight and length (Danaei et al., 2016; Svefors et al., 2019), food security (Black et al., 2013), aflatoxin exposure (Khlangwiset et al., 2011; Smith et al., 2015; Vilcins et al., 2018), environmental enteric dysfunction and hygiene (Humphrey et al., 2015), solid fuel use (Danaei et al., 2016; Vilcins et al., 2018), and women’s time availability and resource control (Johnston et al., 2018; Shroff et al., 2009; Vir, 2016).

Limited information exists about prevalence of non-NDHS stunting determinants in Nigeria, but available evidence indicates that the situation with these determinants is poorer in higher burden zones. Besides, prevalence of undernourishment nearly doubled from 2004–2006 to 2017–2019, and moderate/severe food insecurity increased (Ecker et al., 2020; FAO et al., 2020). In fact, Ecker et al. (2020) report that Nigeria’s chief barrier to improved nutrition is poor diet quality. Apart from unincluded determinants, data limitations also caused our study to use some imprecise indicators. For instance, we measure water and sanitation improvements using drinking water source and open defecation. However, these variables do not capture measures of sanitation such as exposure to animal droppings.

Additional work needs to identify determinants of stunting changes in Nigeria beyond findings in our study, given the magnitude of unexplained stunting reduction, as well as the inability to explain the differences in stunting reduction between Jigawa and Kaduna State. Also, our study assumed stable coefficients across location-specific subpopulations. Other studies (Amare et al., 2018) found significant geopolitical zone differences in the magnitude of coefficients, most affecting zones with the worst NDHS data quality (Fayehun et al., 2020). It is unclear whether coefficient differences are real or a product of poor data quality. Improving data quality of Nigeria surveys is consequently crucial for further efforts to understand and accelerate progress in stunting reduction. Also, southern zones have largely achieved target coverage of stunting predictors included in the projected analysis. Yet, prevalence of stunting in these zones (18% to 25%) is at medium to high levels based on authoritative thresholds (de Onis et al., 2019), emphasizing the importance of location-specific studies.

## Conclusions

Nigeria is a high stunting burden country that is not on track to meet global stunting reduction targets. This paper sought to understand how stunting and stunting determinants in Nigeria have changed over time and explain the slow stunting reductions. Overall, the study found that the limited progress in linear growth and stunting reduction among children under five in Nigeria has been associated with limited improvement in key stunting determinants. Even where improvements have occurred, coverage levels of determinants are still suboptimal. There were wide variations in the levels of determinants and changes in determinant coverage over time across various subpopulations. Our study identified nine multisectoral determinants that could potentially lead to achievement of half of national 2025 stunting reduction goals, if adequately improved. Determinants to be improved are location-specific, signifying the need for nuanced nutrition implementation decisions, rather than templates based on aggregated national data.

## Supplementary Information

Below is the link to the electronic supplementary material.

Supplementary file1 (DOCX 1074 KB)

## Footnotes

1 Although there is data available for 1990 NDHS, this survey was excluded from the analysis because several key determinants, including maternal anthropometric indicators, were not collected as part of that survey. Also, the classification of Nigeria into geopolitical zones had not been done at this time.
