# Supplementary material for: Understanding drivers of stunting reduction in Nigeria from 2003 to 2018: a regression analysis
Source: Food Secur. 2022 Mar 26;14(4):995–1011. doi: 10.1007/s12571-022-01279-8 (PMC9325817; doi:10.1007/s12571-022-01279-8)
Supplement: Supplementary file 1 — Supplementary file1 (DOCX 1074 KB) [file 12571_2022_1279_MOESM1_ESM.docx]

**Journal: *Food Security***

**Understanding Drivers of Stunting Reduction in Nigeria from 2003 to 2018: A Regression Analysis**

Olutayo Adeyemi^1,2^, Mariama Toure^3^, Namukolo Covic^3^, Mara Van Den Bold^3^, Nicholas Nisbett^4^, Derek Headey^3^

^1^Department of Human Nutrition and Dietetics, Faculty of Public Health, University of Ibadan, Ibadan, Nigeria

^3^International Food Policy Research Institute, Washington D.C.

^4^Institute of Development Studies, Brighton, U.K.

^2^Corresponding Author: Email – [adeyemiolutayo@gmail.com](mailto:adeyemiolutayo@gmail.com); ORCID – 0000-0002-5066-517X

**Online Resource 1: Summary of Drivers of Stunting Change Included in Decomposition Analyses**

| **Indicator^a^** | **Description** |
| --- | --- |
| **Outcome Indicators** |  |
| HAZ | Height-for-age z score (HAZ) computed using World Health Organization (WHO) 2006 child growth standards |
| Stunting | HAZ < – 2 SD (Severe stunting = HAZ<–3 SD) |
| **Determinants’ Indicators** |  |
| Child illness | Dummy = 1 if child under five years had diarrhoea or fever or cough in the two weeks preceding the survey |
| Low maternal BMI (0/1) | Dummy = 1 if maternal body mass index (BMI) less than 18.5 kg/m^2^ |
| Maternal height (cm) | Woman’s height in centimetres |
| Exclusive breastfeeding (0/1) | Dummy = 1 if child was fed only breast milk in the 24 hours preceding survey. Computed and included only for children less than 6 (0 to 5) months old |
| Meal frequency (0 – 7) | Number of times child was fed in the 24 hours preceding the survey. In the NDHS data, the values ranged from 0 to 7, with children who were fed more than 7 times assigned 7. Indicator was computed and used for children 6 to 23 months old only |
| Dietary diversity (0 – 6) | Number of food groups child was fed in 24 hours preceding survey. Food groups were classified according to WHO infant and young child feeding guidelines (WHO, 2008). However, the way data was collected in the 2003 NDHS made it impossible to delineate all 7 food groups proffered by WHO. Hence, 6 food groups were used – 1) cereals/tubers; 2) legumes and nuts; 3) dairy; 4) flesh foods and eggs; 5) vitamin A rich fruits and vegetables; 6) other fruits and vegetables. Values for the indicator thus ranged from 0 to 6. Indicator was computed and used for children 6 to 23 months old only |
| Four or more ANC visits (%, village) | Percentage of women in a village that had 4 or more antenatal care (ANC) visits during their last pregnancy. Indicator was used at village level because the information is available only for women who received antenatal care, and only for the last child of each woman. NDHS reports show that more than a third of women in each survey year did not receive any ANC, hence there were many children with missing data. Village performance was considered an adequate proxy for household use given that the indicator at least partially reflects physical access to ANC services (O’Meara et al., 2013) |
| Child born in medical facility (0/1) | Dummy = 1 if child was born in a medical facility |
| Child vaccination (0/1) | Dummy = 1 if child had completed age-appropriate vaccinations. A child was considered fully immunized if the child had received the following vaccinations at the corresponding ages: polio 0 and BCG by 3 months old; previous vaccines, DPT 1 and polio 1 by 4 months old; previous vaccines, DPT 2 and polio 2 by 5 months old; previous vaccines, DPT 3 and polio 3 by 12 months old; previous vaccines and measles by 59 months old |
| Piped water (0/1) | Dummy = 1 if household used a piped drinking water source, including public piped water |
| Borehole/covered well (0/1) | Dummy = 1 if household used borehole, tube well, or covered well drinking water source |
| Open defecation (%, village) | Percentage of households in a village (cluster) that engaged in open defecation. Household members are considered to be less vulnerable to bacteria in their own waste but more susceptible to the bacteria in waste from other households. Village measures of open defecation are therefore preferable to household measures (Headey & Hoddinott, 2015) |
| Asset index (0 – 10) | Index constructed from principal component analysis of 9 variables: radio, television, bicycle, motorcycle, refrigerator, and car ownership, household access to electricity, use of improved floor material, use of improved cooking fuel. The index is re-scaled to vary between 0 and 10 |
| Maternal education (years) | Number of years of maternal education |
| Paternal education (years) | Number of years of paternal education |
| Maternal total fertility | Total number of children ever born by a woman |
| Birth interval (0/1) | Dummy=1 if interval between last child and child preceding last child is less than 18 months. First born children were assigned 0 |
| **Control Indicators** |  |
| Maternal cohort | Dummies for woman’s birth cohort. Birth cohort generated by subtracting the woman’s age from the survey year |
| Maternal age | Dummies for woman’s age in years. Four dummies used. Dummy = 1 if woman’s age <20, ≥20 age <30, ≥30 age <40, ≥40 age <50 or 0 otherwise |
| Child age | Dummies for child’s age in months |
| Child sex | Dummy = 1 if child is a boy |
| Type of place of residence | Dummy = 1 if household resides in rural area |
| Geopolitical zone | Dummies for each geopolitical zone (for national model) |
| Religion | Two dummies were used. Dummy = 1 if household is Christian and dummy = 1 if household practices Islam |
| Marital status | Dummy = 1 if woman is married |
| Survey year | Dummy for survey year |

^a^ Indicators were constructed guided by Headey & Hoddinott (2015); Headey et al. (2015); Headey et al. (2017), except otherwise indicated.

**Online Resource 2: Non-parametric estimates of the relationship between HAZ scores and continuous variables**

**Online Resource 2A: National**

**Online Resource 2B: Rural Areas**

**Online Resource 2C: Urban Areas**

**Online Resource 2D: Children 0 to 5 Months Old**

**Online Resource 2E: Children 6 to 23 Months Old**

**Online Resource 2F: Children 24 to 59 Months Old**

**Online Resource 2G: North Central Zone**

**Online Resource 2H: North East Zone**

**Online Resource 2I: North West Zone**

**Online Resource 2J: South East Zone**

**Online Resource 2K: South South Zone**

**Online Resource 2L: South West Zone**

**Online Resource 3: Checks for multicollinearity**

**Online Resource 3A: Pairwise correlations**

|  | HAZ | Asset index | Maternal education | Paternal education | Number of children | Maternal height | Households with ≥4 ANC visits | Households engaged in open defecation |
| --- | --- | --- | --- | --- | --- | --- | --- | --- |
|  |  |  |  |  |  |  |  |  |
| HAZ | 1.0000 |  |  |  |  |  |  |  |
| Asset index | 0.2253 | 1.0000 |  |  |  |  |  |  |
| Maternal education | 0.2536 | 0.6173 | 1.0000 |  |  |  |  |  |
| Paternal education | 0.1989 | 0.5523 | 0.6469 | 1.0000 |  |  |  |  |
| Maternal total fertility | -0.0862 | -0.1860 | -0.3018 | -0.2065 | 1.0000 |  |  |  |
| Maternal height | 0.1663 | 0.1781 | 0.1805 | 0.1361 | 0.0176 | 1.0000 |  |  |
| Four or more ANC visits (%, village) | 0.2312 | 0.6085 | 0.6129 | 0.5152 | -0.1791 | 0.1590 | 1.0000 |  |
| Open defecation (%, village) | -0.0205 | -0.3319 | -0.0922 | -0.1010 | -0.0159 | -0.0187 | -0.1353 | 1.0000 |

**Online Resource 3B: Variance Inflation Factors (VIF)**

| Variable | VIF | 1/VIF |
| --- | --- | --- |
| Maternal education | 2.58 | 0.387104 |
| Asset index | 2.44 | 0.410248 |
| Four or more ANC visits (%, village) | 2.26 | 0.442045 |
| Paternal education | 1.85 | 0.539526 |
| Child born in medical facility | 1.73 | 0.576384 |
| Child vaccination | 1.30 | 0.769384 |
| Borehole/covered well | 1.22 | 0.820722 |
| Piped water | 1.20 | 0.832161 |
| Open defecation (%, village) | 1.19 | 0.843424 |
| Maternal total fertility | 1.13 | 0.887486 |
| Maternal height | 1.05 | 0.948470 |
| Low maternal BMI | 1.04 | 0.958854 |
| Child illness | 1.02 | 0.983608 |
| Low birth interval | 1.01 | 0.993091 |
|  |  |  |
| Mean VIF | 1.50 |  |

**Online Resource 4: HAZ regressions with dummy variables for categories of open defecation at cluster level**

|  | National | NC | NE | NW | SE | SS | SW |
| --- | --- | --- | --- | --- | --- | --- | --- |
| Low maternal BMI | -0.258*** | -0.224** | -0.199*** | -0.246*** | -0.477*** | -0.297** | -0.352*** |
|  | 0.039 | 0.103 | 0.071 | 0.062 | 0.131 | 0.146 | 0.086 |
| Maternal height (cm) | 0.037*** | 0.049*** | 0.027*** | 0.029*** | 0.050*** | 0.034*** | 0.045*** |
|  | 0.002 | 0.004 | 0.004 | 0.004 | 0.004 | 0.004 | 0.004 |
| Four or more ANC visits | 0.098 | -0.033 | -0.029 | 0.505*** | -0.189 | 0.025 | 0.081 |
|  | 0.069 | 0.134 | 0.165 | 0.129 | 0.22 | 0.169 | 0.182 |
| Delivery in health facility | 0.132*** | 0.177*** | 0.122* | 0.107 | 0.107 | 0.151** | 0.122** |
|  | 0.029 | 0.054 | 0.071 | 0.08 | 0.067 | 0.062 | 0.06 |
| Complete age-appropriate vaccinations | 0.02 | -0.105* | -0.005 | -0.01 | 0.162** | -0.065 | 0.038 |
|  | 0.029 | 0.057 | 0.077 | 0.076 | 0.067 | 0.067 | 0.05 |
| Child illness | -0.155*** | -0.046 | -0.289*** | -0.243*** | -0.127 | -0.066 | -0.029 |
|  | 0.024 | 0.056 | 0.053 | 0.045 | 0.077 | 0.063 | 0.053 |
| Piped water | -0.02 | 0.190** | -0.121 | -0.042 | 0.145 | -0.098 | -0.145* |
|  | 0.042 | 0.093 | 0.084 | 0.082 | 0.124 | 0.096 | 0.083 |
| Borehole/covered well water | -0.024 | 0.027 | 0.077 | -0.042 | -0.05 | -0.031 | -0.129** |
|  | 0.026 | 0.056 | 0.066 | 0.045 | 0.059 | 0.078 | 0.054 |
| Open defecation (>20% of households but ≤40%) | -0.094* | -0.219** | -0.274 | 0.091 | 0.065 | -0.036 | -0.089 |
|  | 0.055 | 0.089 | 0.17 | 0.102 | 0.084 | 0.13 | 0.085 |
| Open defecation (>40% of households but ≤60%) | -0.164*** | -0.114 | -0.173* | -0.157* | 0.106 | -0.358*** | -0.267*** |
|  | 0.045 | 0.094 | 0.104 | 0.087 | 0.106 | 0.116 | 0.098 |
| Open defecation (>60% of households but ≤80%) | -0.126** | -0.156 | -0.213* | 0.085 | 0.205* | -0.371*** | -0.188* |
|  | 0.05 | 0.117 | 0.112 | 0.131 | 0.105 | 0.117 | 0.1 |
| Open defecation (>80% of households but ≤100%) | -0.087* | -0.222** | 0.095 | -0.117 | 0.047 | 0.021 | -0.014 |
|  | 0.046 | 0.09 | 0.118 | 0.104 | 0.092 | 0.119 | 0.083 |
| Asset index (0 – 10) | 0.050*** | 0.044*** | 0.068*** | 0.015 | 0.051*** | 0.033** | 0.079*** |
|  | 0.007 | 0.015 | 0.017 | 0.016 | 0.013 | 0.016 | 0.013 |
| Maternal education (years) | 0.013*** | 0.011* | 0.017** | 0.017*** | 0.006 | 0.023** | 0.009 |
|  | 0.003 | 0.006 | 0.008 | 0.006 | 0.009 | 0.01 | 0.006 |
| Paternal education (years) | 0.005* | 0.009** | -0.011* | 0.005 | 0.013* | 0.021** | 0.007 |
|  | 0.003 | 0.005 | 0.007 | 0.005 | 0.007 | 0.01 | 0.006 |
| Number of children per woman | -0.016** | -0.012 | -0.029* | -0.011 | -0.016 | 0.018 | -0.02 |
|  | 0.007 | 0.015 | 0.017 | 0.012 | 0.016 | 0.022 | 0.017 |
| Low birth interval | -0.150*** | -0.232** | -0.155* | 0.027 | -0.204** | -0.402*** | -0.144 |
|  | 0.045 | 0.097 | 0.085 | 0.083 | 0.09 | 0.152 | 0.113 |
| Male child | -0.202*** | -0.202*** | -0.215*** | -0.257*** | -0.113** | -0.170** | -0.164*** |
|  | 0.022 | 0.045 | 0.041 | 0.043 | 0.048 | 0.073 | 0.043 |
| Rural residence | -0.075** | 0.055 | -0.133 | -0.146** | 0.028 | -0.111 | -0.076 |
|  | 0.035 | 0.074 | 0.085 | 0.073 | 0.067 | 0.072 | 0.068 |
| Year 2008 | 0.170*** | -0.220** | -0.04 | 0.641*** | 0.129 | -0.195 | 0.208* |
|  | 0.054 | 0.107 | 0.112 | 0.09 | 0.159 | 0.139 | 0.116 |
| Year 2013 | 0.355*** | 0.341** | 0.258* | 0.371*** | 0.330* | 0.418** | 0.431*** |
|  | 0.069 | 0.144 | 0.155 | 0.116 | 0.196 | 0.194 | 0.146 |
| Year 2018 | 0.160* | 0.152 | -0.054 | 0.343** | 0.145 | 0.014 | 0.176 |
|  | 0.085 | 0.173 | 0.187 | 0.146 | 0.236 | 0.23 | 0.185 |
| R-squared | 0.209 | 0.186 | 0.183 | 0.159 | 0.175 | 0.181 | 0.178 |
| N | 57507 | 9752 | 11969 | 15306 | 5794 | 6778 | 7908 |

Note: Clustered robust standard errors are below point estimates. ***, ** and * indicate significance at 1%, 5%, and 10% levels, respectively. The regressions included several time-invariant controls, including zonal fixed effects, dummy variables for practice of Christianity and Islam, month-specific child age dummy variables, and dummy variables for various categories of maternal age and maternal cohort.

**Online Resource 5: Nutrition Outcomes and Nutrition Determinants in NDHS 2003, 2008, 2013, and 2018**

|  | **National** | **Rural** | **Urban** | **North Central** | **North East** | **North West** | **South East** | **South South** | **South West** | **0 – 5 Months Old** | **6 – 23 Months Old** | **24 – 59 Months Old** |
| --- | --- | --- | --- | --- | --- | --- | --- | --- | --- | --- | --- | --- |
| **Mean HAZ** |  |  |  |  |  |  |  |  |  |  |  |  |
| 2003 | -1.62 | -1.79 | -1.26 | -1.28 | -1.82 | -2.37 | -0.66 | -0.77 | -1.14 | -0.24 | -1.57 | -1.96 |
| 2008 | -1.47 | -1.67 | -1.05 | -1.66 | -1.77 | -1.91 | -0.69 | -1.13 | -1.10 | -0.28 | -1.40 | -1.72 |
| 2013 | -1.37 | -1.63 | -0.94 | -1.05 | -1.47 | -2.18 | -0.46 | -0.52 | -0.81 | -0.03 | -1.14 | -1.73 |
| 2018 | -1.52 | -1.80 | -1.17 | -1.20 | -1.90 | -2.21 | -0.84 | -0.99 | -1.13 | -0.81 | -1.40 | -1.72 |
| **% Stunting (HAZ< – 2)** |  |  |  |  |  |  |  |  |  |  |  |  |
| 2003 | 42.56 | 47.56 | 31.81 | 36.24 | 47.40 | 60.21 | 18.25 | 22.54 | 28.88 | 16.98 | 40.83 | 49.22 |
| 2008 | 40.56 | 45.09 | 31.03 | 43.38 | 48.90 | 52.82 | 21.08 | 30.63 | 30.73 | 21.43 | 40.60 | 43.92 |
| 2013 | 36.76 | 43.11 | 26.00 | 28.42 | 42.35 | 54.92 | 15.45 | 18.07 | 21.72 | 15.60 | 33.34 | 42.25 |
| 2018 | 36.54 | 44.58 | 26.41 | 27.87 | 48.71 | 56.79 | 18.13 | 19.88 | 22.86 | 18.47 | 33.38 | 41.60 |
| **% Severe Stunting (HAZ< – 3)** |  |  |  |  |  |  |  |  |  |  |  |  |
| 2003 | 22.79 | 26.32 | 15.21 | 13.89 | 24.86 | 40.11 | 5.91 | 7.37 | 9.69 | 6.85 | 21.21 | 27.24 |
| 2008 | 22.78 | 26.36 | 15.22 | 25.00 | 28.97 | 33.86 | 8.61 | 13.88 | 13.41 | 9.82 | 23.24 | 24.82 |
| 2013 | 21.07 | 25.81 | 13.04 | 13.97 | 23.81 | 36.21 | 5.30 | 8.04 | 7.99 | 7.69 | 19.17 | 24.40 |
| 2018 | 16.95 | 22.52 | 9.93 | 9.98 | 25.58 | 30.67 | 5.26 | 4.78 | 8.17 | 6.43 | 13.06 | 21.05 |
| **% Underweight (BMI<18.5 kg/m^2^)** |  |  |  |  |  |  |  |  |  |  |  |  |
| 2003 | 12.32 | 13.85 | 9.01 | 6.16 | 21.07 | 14.71 | 4.68 | 5.10 | 8.69 | 10.51 | 14.44 | 11.44 |
| 2008 | 11.14 | 13.01 | 7.19 | 7.27 | 18.48 | 16.43 | 4.82 | 5.50 | 7.27 | 10.39 | 12.90 | 10.31 |
| 2013 | 8.18 | 9.65 | 5.71 | 4.97 | 12.77 | 10.59 | 3.13 | 4.19 | 6.25 | 7.34 | 9.84 | 7.41 |
| 2018 | 9.39 | 12.08 | 6.01 | 6.78 | 19.46 | 12.43 | 3.06 | 3.28 | 5.96 | 7.88 | 11.06 | 8.73 |
| **Average height (cm)** |  |  |  |  |  |  |  |  |  |  |  |  |
| 2003 | 158.39 | 157.81 | 159.64 | 158.29 | 157.97 | 157.59 | 159.86 | 159.13 | 160.05 | 158.24 | 158.40 | 158.42 |
| 2008 | 158.05 | 157.48 | 159.28 | 158.02 | 157.57 | 156.44 | 159.76 | 158.61 | 159.58 | 157.67 | 157.90 | 158.21 |
| 2013 | 158.45 | 157.75 | 159.63 | 158.38 | 158.26 | 157.17 | 160.98 | 158.73 | 159.91 | 158.02 | 158.32 | 158.59 |
| 2018 | 158.43 | 157.70 | 159.35 | 158.43 | 158.35 | 157.28 | 159.94 | 158.40 | 159.31 | 157.80 | 158.46 | 158.52 |
| **% Child Illness in 2 Weeks Preceding Survey** |  |  |  |  |  |  |  |  |  |  |  |  |
| 2003 | 46.81 | 49.88 | 40.24 | 40.05 | 63.73 | 44.74 | 38.62 | 47.01 | 30.28 | 37.20 | 57.11 | 42.78 |
| 2008 | 26.87 | 28.29 | 23.86 | 17.33 | 36.62 | 26.24 | 32.48 | 33.80 | 18.43 | 20.86 | 34.68 | 23.71 |
| 2013 | 23.05 | 22.90 | 23.31 | 17.40 | 37.16 | 17.81 | 31.61 | 25.87 | 17.90 | 15.01 | 32.10 | 19.37 |
| 2018 | 36.58 | 40.78 | 31.28 | 31.37 | 54.19 | 41.26 | 32.82 | 39.66 | 18.76 | 26.18 | 44.07 | 34.22 |
| **ANC at Least Four Visits, % Households in Village** |  |  |  |  |  |  |  |  |  |  |  |  |
| 2003 | 51.46 | 40.32 | 75.36 | 57.89 | 34.33 | 31.14 | 84.63 | 71.30 | 95.32 | 49.33 | 51.74 | 51.76 |
| 2008 | 53.46 | 41.86 | 77.91 | 51.98 | 34.14 | 26.77 | 79.50 | 64.13 | 89.02 | 52.93 | 53.29 | 53.64 |
| 2013 | 54.19 | 40.08 | 78.13 | 55.96 | 42.57 | 30.95 | 86.73 | 68.46 | 89.50 | 53.31 | 54.77 | 54.02 |
| 2018 | 61.79 | 48.74 | 78.24 | 56.07 | 44.35 | 42.87 | 83.95 | 72.95 | 89.93 | 59.81 | 61.80 | 62.14 |
| **% Delivery in Health Facility** |  |  |  |  |  |  |  |  |  |  |  |  |
| 2003 | 36.61 | 26.59 | 58.11 | 47.02 | 18.20 | 12.40 | 88.52 | 54.30 | 78.90 | 34.34 | 37.86 | 36.37 |
| 2008 | 38.05 | 27.21 | 60.91 | 43.19 | 13.74 | 9.86 | 75.37 | 46.89 | 70.71 | 38.69 | 37.42 | 38.28 |
| 2013 | 38.17 | 23.31 | 63.37 | 46.52 | 21.24 | 12.05 | 79.63 | 50.57 | 75.47 | 37.25 | 39.92 | 37.36 |
| 2018 | 45.58 | 29.94 | 65.29 | 50.27 | 26.18 | 17.15 | 83.50 | 48.91 | 75.91 | 44.53 | 46.38 | 45.31 |
| **% Children Fully Vaccinated at Appropriate Age** |  |  |  |  |  |  |  |  |  |  |  |  |
| 2003 | 11.68 | 7.16 | 21.39 | 14.27 | 4.65 | 0.78 | 24.41 | 15.04 | 44.12 | 11.61 | 11.69 | 11.69 |
| 2008 | 25.95 | 17.66 | 43.42 | 24.47 | 6.75 | 5.72 | 51.46 | 36.45 | 52.26 | 25.20 | 26.14 | 25.98 |
| 2013 | 30.28 | 18.01 | 51.10 | 33.36 | 14.94 | 11.36 | 65.83 | 48.35 | 54.40 | 24.68 | 31.04 | 30.82 |
| 2018 | 23.99 | 16.60 | 33.31 | 22.57 | 16.27 | 12.54 | 40.32 | 32.88 | 33.59 | 35.93 | 39.97 | 12.78 |
| **% Piped Drinking Water Source** |  |  |  |  |  |  |  |  |  |  |  |  |
| 2003 | 15.53 | 7.26 | 33.29 | 13.34 | 12.14 | 20.56 | 16.08 | 6.71 | 21.88 | 13.44 | 16.28 | 15.54 |
| 2008 | 9.88 | 5.20 | 19.76 | 8.81 | 6.08 | 10.72 | 5.27 | 10.69 | 14.48 | 9.53 | 10.00 | 9.89 |
| 2013 | 9.06 | 5.39 | 15.28 | 7.75 | 6.88 | 9.75 | 6.53 | 9.73 | 12.23 | 9.04 | 9.44 | 8.85 |
| 2018 | 10.81 | 9.52 | 12.44 | 6.52 | 14.31 | 13.32 | 3.05 | 18.93 | 7.93 | 12.23 | 10.51 | 10.73 |
| **% Borehole/Covered Well Drinking Water Source** |  |  |  |  |  |  |  |  |  |  |  |  |
| 2003 | 21.59 | 17.82 | 29.70 | 17.77 | 7.33 | 15.22 | 56.59 | 39.42 | 33.52 | 22.76 | 21.69 | 21.28 |
| 2008 | 39.88 | 34.20 | 51.86 | 36.09 | 26.32 | 35.89 | 53.98 | 40.45 | 52.22 | 40.06 | 39.54 | 40.04 |
| 2013 | 46.72 | 39.42 | 59.10 | 43.05 | 38.61 | 45.01 | 58.21 | 53.59 | 51.66 | 47.11 | 45.92 | 47.11 |
| 2018 | 48.46 | 44.23 | 53.79 | 53.04 | 35.56 | 41.94 | 63.46 | 45.01 | 57.85 | 47.12 | 47.87 | 49.03 |
| **Open Defecation, % Households in Village** |  |  |  |  |  |  |  |  |  |  |  |  |
| 2003 | 24.53 | 32.02 | 8.46 | 41.55 | 18.36 | 17.11 | 19.77 | 32.27 | 28.58 | 21.83 | 24.45 | 25.17 |
| 2008 | 30.41 | 39.00 | 12.31 | 57.31 | 30.85 | 15.47 | 20.27 | 31.23 | 37.49 | 29.96 | 30.70 | 30.34 |
| 2013 | 29.47 | 37.95 | 15.11 | 58.45 | 26.45 | 15.63 | 33.00 | 30.28 | 34.60 | 31.56 | 28.81 | 29.49 |
| 2018 | 23.45 | 30.15 | 14.99 | 51.14 | 20.18 | 9.26 | 27.00 | 23.24 | 24.83 | 22.82 | 23.80 | 23.36 |
| **Mean Asset Index** |  |  |  |  |  |  |  |  |  |  |  |  |
| 2003 | 3.53 | 2.42 | 5.90 | 3.24 | 2.19 | 3.00 | 5.82 | 4.31 | 6.10 | 3.58 | 3.49 | 3.54 |
| 2008 | 3.47 | 2.38 | 5.76 | 2.89 | 1.95 | 2.51 | 4.52 | 4.23 | 5.49 | 3.39 | 3.41 | 3.51 |
| 2013 | 3.61 | 2.37 | 5.72 | 3.49 | 2.37 | 2.62 | 4.59 | 4.93 | 5.92 | 3.54 | 3.61 | 3.62 |
| 2018 | 4.08 | 2.81 | 5.69 | 3.87 | 2.44 | 2.83 | 5.18 | 5.40 | 6.16 | 4.11 | 4.07 | 4.08 |
| **Mean Maternal Education (years)** |  |  |  |  |  |  |  |  |  |  |  |  |
| 2003 | 4.29 | 3.15 | 6.72 | 4.32 | 2.26 | 2.28 | 8.62 | 7.32 | 8.29 | 4.33 | 4.30 | 4.27 |
| 2008 | 5.09 | 3.80 | 7.80 | 4.73 | 2.04 | 1.91 | 8.82 | 8.27 | 8.36 | 5.22 | 5.17 | 5.01 |
| 2013 | 4.94 | 3.12 | 8.02 | 5.10 | 2.60 | 1.96 | 9.29 | 8.75 | 9.09 | 4.97 | 5.24 | 4.76 |
| 2018 | 6.28 | 3.96 | 9.20 | 6.15 | 3.06 | 2.48 | 10.43 | 10.09 | 10.17 | 6.40 | 6.44 | 6.16 |
| **Mean Paternal Education (years)** |  |  |  |  |  |  |  |  |  |  |  |  |
| 2003 | 5.85 | 4.77 | 8.16 | 6.94 | 4.29 | 4.29 | 7.83 | 7.80 | 8.70 | 5.61 | 6.01 | 5.80 |
| 2008 | 6.27 | 5.03 | 8.88 | 6.66 | 3.41 | 3.79 | 8.03 | 9.00 | 9.19 | 6.32 | 6.36 | 6.22 |
| 2013 | 6.34 | 4.65 | 9.22 | 7.95 | 4.26 | 3.73 | 8.74 | 9.36 | 9.71 | 6.29 | 6.54 | 6.24 |
| 2018 | 7.41 | 5.48 | 9.85 | 7.81 | 5.16 | 4.67 | 9.56 | 10.24 | 10.31 | 7.60 | 7.43 | 7.37 |
| **Mean Number of Children per Woman** |  |  |  |  |  |  |  |  |  |  |  |  |
| 2003 | 4.38 | 4.50 | 4.12 | 4.06 | 4.82 | 4.62 | 4.00 | 4.42 | 3.34 | 3.85 | 4.06 | 4.68 |
| 2008 | 4.30 | 4.52 | 3.84 | 4.12 | 4.88 | 4.85 | 4.08 | 4.12 | 3.35 | 3.91 | 3.97 | 4.54 |
| 2013 | 4.30 | 4.50 | 3.95 | 3.88 | 4.68 | 4.88 | 4.01 | 3.80 | 3.43 | 4.00 | 3.90 | 4.57 |
| 2018 | 4.20 | 4.52 | 3.78 | 3.91 | 4.70 | 5.10 | 3.77 | 3.55 | 3.19 | 3.86 | 3.86 | 4.45 |
| **% Birth Interval Less than 18 Months** |  |  |  |  |  |  |  |  |  |  |  |  |
| 2003 | 6.33 | 6.62 | 5.69 | 4.48 | 7.36 | 6.43 | 8.73 | 7.08 | 3.92 | 4.40 | 5.33 | 7.34 |
| 2008 | 5.55 | 5.48 | 5.69 | 4.19 | 5.26 | 5.80 | 8.93 | 6.50 | 3.94 | 3.72 | 5.62 | 5.84 |
| 2013 | 5.02 | 4.96 | 5.13 | 3.96 | 5.62 | 4.87 | 8.09 | 4.78 | 3.98 | 3.42 | 4.12 | 5.79 |
| 2018 | 6.03 | 6.00 | 6.06 | 5.30 | 6.05 | 6.10 | 7.96 | 6.24 | 4.97 | 4.27 | 4.29 | 7.33 |
| **% Received 0 Health Sector Continuum of Care Actions** |  |  |  |  |  |  |  |  |  |  |  |  |
| 2003 | 48.78 | 59.18 | 26.47 | 34.69 | 66.86 | 73.11 | 5.88 | 30.56 | 7.08 | 45.74 | 40.85 | 54.18 |
| 2008 | 46.33 | 57.70 | 22.35 | 40.41 | 69.31 | 77.80 | 13.22 | 32.54 | 11.90 | 40.18 | 41.12 | 50.24 |
| 2013 | 43.73 | 58.58 | 18.57 | 35.14 | 58.26 | 70.18 | 6.45 | 25.28 | 9.84 | 40.27 | 36.93 | 48.11 |
| 2018 | 35.94 | 49.89 | 18.36 | 34.69 | 52.40 | 58.80 | 8.13 | 28.04 | 9.93 | 29.02 | 26.42 | 42.58 |
| **% Received 3 Health Sector Continuum of Care Actions** |  |  |  |  |  |  |  |  |  |  |  |  |
| 2003 | 6.33 | 3.59 | 12.21 | 6.42 | 2.40 | 0.39 | 10.22 | 8.64 | 27.75 | 8.04 | 8.33 | 4.76 |
| 2008 | 10.22 | 5.69 | 19.77 | 9.40 | 1.89 | 1.52 | 17.53 | 11.59 | 25.95 | 13.11 | 14.61 | 7.34 |
| 2013 | 13.44 | 6.34 | 25.48 | 14.68 | 4.16 | 2.91 | 32.85 | 18.98 | 31.18 | 14.91 | 19.53 | 9.80 |
| 2018 | 12.17 | 6.25 | 19.64 | 11.06 | 5.53 | 3.28 | 25.59 | 16.43 | 21.23 | 19.16 | 22.07 | 5.31 |
| **% Received 0 Multisectoral Interventions** |  |  |  |  |  |  |  |  |  |  |  |  |
| 2003 | 6.90 | 10.04 | 0.15 | 6.96 | 9.56 | 10.13 | 0.76 | 1.56 | 1.65 | 6.07 | 6.06 | 7.58 |
| 2008 | 7.04 | 10.02 | 0.76 | 11.22 | 15.57 | 7.32 | 1.53 | 1.85 | 3.02 | 6.44 | 6.38 | 7.50 |
| 2013 | 6.99 | 10.95 | 0.28 | 11.10 | 12.32 | 7.54 | 0.60 | 1.64 | 3.33 | 7.43 | 5.71 | 7.63 |
| 2018 | 3.94 | 6.72 | 0.43 | 8.19 | 8.29 | 3.94 | 0.38 | 0.64 | 1.24 | 3.07 | 3.63 | 4.26 |
| **% Received 6 Multisectoral Interventions** |  |  |  |  |  |  |  |  |  |  |  |  |
| 2003 | 3.28 | 1.61 | 6.86 | 2.94 | 0.28 | 0.22 | 8.47 | 5.69 | 13.41 | 4.97 | 4.26 | 2.32 |
| 2008 | 5.61 | 2.16 | 12.87 | 4.30 | 0.57 | 1.00 | 9.88 | 7.65 | 13.93 | 7.18 | 8.15 | 3.95 |
| 2013 | 7.22 | 2.57 | 15.10 | 6.60 | 2.66 | 2.19 | 16.09 | 12.21 | 15.73 | 8.77 | 10.05 | 5.38 |
| 2018 | 6.44 | 3.08 | 10.69 | 5.12 | 3.20 | 1.98 | 14.52 | 8.97 | 10.33 | 9.73 | 11.60 | 2.93 |

**Online Resource 6A: HAZ and stunting regressions pooled across 2003, 2008, 2013 and 2018 NDHS; with number of 6 selected multisectoral drivers received (discrete variable) replacing individual driver indicators**

|  | Full National Sample, HAZ OLS | Full National Sample, Stunting LPM | Full National Sample, Severe Stunting LPM |
| --- | --- | --- | --- |
| Number of multisectoral drivers received | 0.042*** | -0.013*** | -0.013*** |
|  | 0.01 | 0.002 | 0.002 |
| Low maternal BMI | -0.276*** | 0.066*** | 0.041*** |
|  | 0.039 | 0.01 | 0.009 |
| Maternal height (cm) | 0.037*** | -0.008*** | -0.005*** |
|  | 0.002 | 0 | 0 |
| Child illness | -0.157*** | 0.026*** | 0.032*** |
|  | 0.025 | 0.006 | 0.005 |
| Asset index (1 – 10) | 0.069*** | -0.018*** | -0.010*** |
|  | 0.007 | 0.001 | 0.001 |
| Number of children per woman | -0.024*** | 0.005*** | 0.001 |
|  | 0.007 | 0.002 | 0.001 |
| Low birth interval | -0.143*** | 0.040*** | 0.039*** |
|  | 0.045 | 0.012 | 0.011 |
| Male child | -0.201*** | 0.052*** | 0.035*** |
|  | 0.022 | 0.005 | 0.005 |
| Rural residence | -0.100*** | 0.025*** | 0.019*** |
|  | 0.034 | 0.007 | 0.005 |
| Year 2008 | 0.165*** | -0.017 | 0.007 |
|  | 0.054 | 0.011 | 0.01 |
| Year 2013 | 0.346*** | -0.072*** | -0.023* |
|  | 0.069 | 0.015 | 0.013 |
| Year 2018 | 0.177** | -0.060*** | -0.055*** |
|  | 0.085 | 0.02 | 0.017 |
| R-squared | 0.207 | 0.175 | 0.137 |
| N | 57507 | 57507 | 57507 |

Note: Clustered robust standard errors are below point estimates. ***, ** and * indicate significance at 1%, 5%, and 10% levels, respectively. The regressions included several time-invariant controls, including zonal fixed effects, dummy variables for practice of Christianity and Islam, month-specific child age dummy variables, and dummy variables for various categories of maternal age and maternal cohort.

**Online Resource 6B: HAZ and stunting regressions pooled across 2003, 2008, 2013 and 2018 NDHS with number of 6 selected multisectoral drivers received (categorical variable) replacing individual driver indicators**

|  | Full National Sample, HAZ OLS | Full National Sample, Stunting LPM | Full National Sample, Severe Stunting LPM |
| --- | --- | --- | --- |
| 0 multisectoral drivers received | 0 | 0 | 0 |
| 1 multisectoral drivers received | -0.100* | 0.019 | 0.005 |
|  | 0.056 | 0.013 | 0.012 |
| 2 multisectoral drivers received | -0.004 | 0.006 | -0.020* |
|  | 0.056 | 0.013 | 0.012 |
| 3 multisectoral drivers received | 0.028 | -0.012 | -0.033*** |
|  | 0.061 | 0.013 | 0.012 |
| 4 multisectoral drivers received | 0.105* | -0.041*** | -0.058*** |
|  | 0.061 | 0.014 | 0.013 |
| 5 multisectoral drivers received | 0.141** | -0.052*** | -0.057*** |
|  | 0.064 | 0.015 | 0.013 |
| 6 multisectoral drivers received | 0.130* | -0.036** | -0.049*** |
|  | 0.075 | 0.016 | 0.014 |
| Low maternal BMI | -0.278*** | 0.066*** | 0.041*** |
|  | 0.039 | 0.01 | 0.009 |
| Maternal height (cm) | 0.037*** | -0.008*** | -0.005*** |
|  | 0.002 | 0 | 0 |
| Child illness | -0.157*** | 0.026*** | 0.033*** |
|  | 0.025 | 0.006 | 0.005 |
| Asset index (1 – 10) | 0.068*** | -0.018*** | -0.010*** |
|  | 0.007 | 0.001 | 0.001 |
| Number of children per woman | -0.023*** | 0.005*** | 0.001 |
|  | 0.007 | 0.002 | 0.001 |
| Low birth interval | -0.140*** | 0.039*** | 0.038*** |
|  | 0.045 | 0.012 | 0.011 |
| Male child | -0.201*** | 0.052*** | 0.035*** |
|  | 0.022 | 0.005 | 0.005 |
| Rural residence | -0.099*** | 0.024*** | 0.019*** |
|  | 0.034 | 0.007 | 0.005 |
| Year 2008 | 0.163*** | -0.016 | 0.006 |
|  | 0.054 | 0.011 | 0.01 |
| Year 2013 | 0.342*** | -0.071*** | -0.024* |
|  | 0.069 | 0.015 | 0.013 |
| Year 2018 | 0.174** | -0.059*** | -0.056*** |
|  | 0.084 | 0.02 | 0.017 |
| R-squared | 0.207 | 0.176 | 0.137 |
| N | 57507 | 57507 | 57507 |

Note: Clustered robust standard errors are below point estimates. ***, ** and * indicate significance at 1%, 5%, and 10% levels, respectively. The regressions included several time-invariant controls, including zonal fixed effects, dummy variables for practice of Christianity and Islam, month-specific child age dummy variables, and dummy variables for various categories of maternal age and maternal cohort.

**Online Resource 7A: Decomposition of 2003 to 2018 changes in stunting and severe stunting prevalence in Nigeria**

|  | Actual change from 2003 to 2018  (A) | **Stunting** | | | **Severe stunting** | | |
| --- | --- | --- | --- | --- | --- | --- | --- |
|  |  | Estimated β  (B) | Predicted change  (C = A x B) | Predicted change as a share of actual change in stunting  (D=(C/-0.060)*100) | Estimated β  (E) | Predicted change  (F=A x E) | Predicted change as a share of actual change in severe stunting  (G=(F/-0.058)*100) |
| Stunting | -0.060 |  | -0.030 | ***49.53*** |  |  |  |
| Severe stunting | -0.058 |  |  |  |  | -0.026 | ***44.57*** |
| Low maternal BMI | -0.029 | 0.062 | -0.002 | 3.01 | 0.038 | -0.001 | 1.90 |
| Maternal height | 0.036 | -0.008 | 0.000 | 0.48 | -0.005 | 0.000 | 0.31 |
| Four or more ANC visits | 0.103 |  |  |  | -0.068 | -0.007 | 11.85 |
| Delivery in health facility | 0.090 | -0.039 | -0.003 | 5.81 | -0.015 | -0.001 | 2.30 |
| Child illness | -0.082 | 0.034 | -0.003 | 4.42 | 0.035 | -0.003 | 5.78 |
| Asset index | 0.555 | -0.015 | -0.008 | 13.84 | -0.007 | -0.004 | 6.65 |
| Maternal education | 1.989 | -0.005 | -0.010 | 16.54 | -0.003 | -0.006 | 10.22 |
| Paternal education | 1.564 | -0.002 | -0.003 | 5.20 | -0.002 | -0.003 | 5.36 |
| Low birth interval | -0.003 | 0.043 | 0.000 | 0.21 | 0.039 | 0.000 | 0.20 |

**Online Resource 7B: Decomposition of 2008 to 2013 changes in HAZ, stunting, and severe stunting prevalence in Nigeria**

|  | Change from 2008 to 2013 | **HAZ** | | | **Stunting** | | | **Severe Stunting** | | |
| --- | --- | --- | --- | --- | --- | --- | --- | --- | --- | --- |
|  |  | Estimated β | Predicted HAZ change | Share of actual change (%) | Estimated β | Predicted stunting change | Share of actual change (%) | Estimated β | Predicted severe stunting change | Share of actual change (%) |
| HAZ | 0.100 |  | 0.036 | ***35.88*** |  |  |  |  |  |  |
| Stunting | -0.038 |  |  |  |  | -0.008 | ***20.44*** |  |  |  |
| Severe stunting | -0.017 |  |  |  |  |  |  |  | -0.006 | ***33.72*** |
| Low maternal BMI | -0.030 | -0.265 | 0.008 | 7.80 | 0.062 | -0.002 | 4.81 | 0.038 | -0.001 | 6.57 |
| Maternal height | 0.393 | 0.037 | 0.015 | 14.50 | -0.008 | -0.003 | 8.26 | -0.005 | -0.002 | 11.51 |
| Four or more ANC visits | 0.007 |  |  |  |  |  |  | -0.067 | 0.000 | 2.89 |
| Delivery in health facility | 0.001 | 0.146 | 0.000 | 0.17 | -0.039 | 0.000 | 0.12 | -0.015 | 0.000 | 0.11 |
| Child illness | -0.038 | -0.156 | 0.006 | 5.94 | 0.026 | -0.001 | 2.61 | 0.033 | -0.001 | 7.38 |
| Open defecation | -0.009 | -0.128 | 0.001 | 1.20 |  | 0.000 | 0.00 |  |  |  |
| Asset index | 0.142 | 0.055 | 0.008 | 7.82 | -0.015 | -0.002 | 5.62 | -0.007 | -0.001 | 5.84 |
| Maternal education | -0.148 | 0.016 | -0.002 | -2.37 | -0.005 | 0.001 | -1.95 | -0.003 | 0.000 | -2.61 |
| Paternal education | 0.070 |  |  |  | -0.002 | 0.000 | 0.37 | -0.002 | 0.000 | 0.83 |
| Number of children per woman | -0.002 | -0.017 | 0.000 | 0.03 |  |  |  |  |  |  |
| Low birth interval | -0.005 | -0.149 | 0.001 | 0.79 | 0.043 | 0.000 | 0.60 | 0.039 | 0.000 | 1.21 |

**Online Resource 8: Rural/urban differences in stunting reduction**

The improvements in stunting observed at the national level appeared to occur mostly in urban areas (Online Resource 8a). Rural areas did not see a statistically significant decline in stunting or severe stunting from 2003 to 2018, while urban areas had significant declines in both outcomes (p<0.05). Although rural areas had greater 2003 to 2018 improvements in several determinants (including vaccination, ≥4 ANC visits, and borehole drinking water source), the 2018 coverage of determinants in rural areas was still not at par with urban coverage (Online Resource 8a).

Factors associated with HAZ, stunting, and severe stunting, were similar for rural and urban areas (Online Resource 8b), as with the full sample, with a few notable exceptions. Child vaccinations, open defecation, and low birth intervals were significant in urban but not rural areas; while low maternal BMI, child illnesses, health facility delivery, and paternal education were significant in rural but not urban areas. Decomposition analysis was conducted only for urban areas, given that there had been no change in rural areas. Improved maternal education was the most important contributor to predicted stunting progress. The predictive ability of the model was reduced for both outcomes because asset index and maternal height declined, and low birth interval increased, from 2003 to 2018 (Online Resource 8c).

**Online Resource 8A: Nutrition outcomes and determinants of nutrition for children 0 to 59 months old in 2003 and 2018, in rural and urban areas**

| **Characteristics** | **Rural** | | | **Urban** | | |
| --- | --- | --- | --- | --- | --- | --- |
|  | **2003** | **2018** | **% ∆** | **2003** | **2018** | **% ∆** |
| ***Outcomes*** |  |  |  |  |  |  |
| Mean HAZ | -1.79 | -1.80 | *0.44* | -1.26 | -1.17 | *-6.94* |
| % Stunting (HAZ< – 2) | 47.56 | 44.58 | *-6.28* | 31.81 | 26.41 | *-16.97*** |
| % Severe Stunting (HAZ< – 3) | 26.32 | 22.52 | *-14.44** | 15.21 | 9.93 | *-34.76**** |
| ***Maternal Nutrition*** |  |  |  |  |  |  |
| % Underweight (BMI<18.5 kg/m^2^) | 13.85 | 12.08 | *-12.81* | 9.01 | 6.01 | *-33.32*** |
| Average height (cm) | 157.81 | 157.70 | *-0.07* | 159.64 | 159.35 | *-0.18* |
| ***Health and Health Seeking*** |  |  |  |  |  |  |
| % ANC at Least Four Visits | 40.32 | 48.74 | *20.88**** | 75.36 | 78.24 | *3.81* |
| % Delivery in Health Facility | 26.59 | 29.94 | *12.57* | 58.11 | 65.29 | *12.36* |
| % Children Fully Vaccinated at Appropriate Age | 7.16 | 16.60 | *131.86**** | 21.39 | 33.31 | *55.69**** |
| % Child Illness in 2 Weeks Preceding Survey | 49.88 | 40.78 | *-18.23**** | 40.24 | 31.28 | *-22.26**** |
| ***Water and Sanitation*** |  |  |  |  |  |  |
| % Piped Drinking Water Source | 7.26 | 9.52 | *31.24* | 33.29 | 12.44 | *-62.63**** |
| % Borehole/Covered Well Drinking Water Source | 17.82 | 44.23 | *148.28**** | 29.70 | 53.79 | *81.09**** |
| % Households with Open Defecation | 32.02 | 30.15 | *-5.82* | 8.46 | 14.99 | *77.26*** |
| ***Wealth and Education*** |  |  |  |  |  |  |
| Mean Asset Index | 2.42 | 2.81 | *15.82*** | 5.90 | 5.69 | *-3.51* |
| Mean Maternal Education (years) | 3.15 | 3.96 | *25.43*** | 6.72 | 9.20 | *36.93**** |
| Mean Paternal Education (years) | 4.77 | 5.48 | *14.82*** | 8.16 | 9.85 | *20.74**** |
| ***Demography*** |  |  |  |  |  |  |
| Mean Number of Children per Woman | 4.50 | 4.52 | *0.60* | 4.12 | 3.78 | *-8.19*** |
| % Birth Interval Less than 18 Months | 6.62 | 6.00 | *-9.37* | 5.69 | 6.06 | *6.55* |
| ***Enabling Environment*** |  |  |  |  |  |  |
| % Received 0 Health Sector Continuum of Care Actions | 59.18 | 49.89 | *-15.70**** | 26.47 | 18.36 | *-30.64*** |
| % Received 3 Health Sector Continuum of Care Actions | 3.59 | 6.25 | *74.15*** | 12.21 | 19.64 | *60.85**** |
| % Received 0 of 6 Multisectoral Drivers | 10.04 | 6.72 | *-33.10** | 0.15 | 0.43 | *182.13* |
| % Received 6 of 6 Multisectoral Drivers | 1.61 | 3.08 | *91.63* | 6.86 | 10.69 | *55.70**** |

***, ** and * indicate significance at 1%, 5%, and 10% levels, respectively

**Online Resource 8B: HAZ and stunting regressions pooled across 2003, 2008, 2013 and 2018 NDHS for rural and urban areas**

|  | Rural sample HAZ | Urban sample HAZ | Rural sample stunting | Urban sample stunting | Rural sample severe stunting | Urban sample severe stunting |
| --- | --- | --- | --- | --- | --- | --- |
| Low maternal BMI | -0.238*** | -0.315*** | 0.057*** | 0.075*** | 0.046*** | 0.011 |
|  | 0.045 | 0.074 | 0.011 | 0.021 | 0.011 | 0.014 |
| Maternal height (cm) | 0.035*** | 0.041*** | -0.008*** | -0.009*** | -0.005*** | -0.005*** |
|  | 0.002 | 0.003 | 0.001 | 0.001 | 0.001 | 0.001 |
| Four or more ANC visits | 0.034 | 0.254* | 0.030* | -0.035 | -0.036*** | -0.083*** |
|  | 0.084 | 0.139 | 0.016 | 0.028 | 0.014 | 0.023 |
| Delivery in health facility | 0.150*** | 0.102** | -0.040*** | -0.032*** | -0.027*** | -0.001 |
|  | 0.037 | 0.043 | 0.01 | 0.011 | 0.008 | 0.008 |
| Complete age-appropriate vaccinations | 0.045 | 0.005 | -0.014 | -0.016* | -0.009 | -0.017** |
|  | 0.042 | 0.039 | 0.009 | 0.009 | 0.007 | 0.006 |
| Child illness | -0.207*** | -0.075* | 0.034*** | 0.011 | 0.048*** | 0.003 |
|  | 0.031 | 0.039 | 0.008 | 0.01 | 0.007 | 0.008 |
| Piped water | -0.032 | -0.046 | 0.01 | 0.004 | 0.005 | 0.001 |
|  | 0.063 | 0.057 | 0.015 | 0.013 | 0.014 | 0.011 |
| Borehole/covered well water | -0.008 | -0.052 | -0.005 | -0.004 | 0.003 | -0.009 |
|  | 0.032 | 0.042 | 0.007 | 0.009 | 0.006 | 0.007 |
| Open defecation | -0.098 | -0.212*** | 0.013 | 0.021 | 0.017* | -0.005 |
|  | 0.061 | 0.067 | 0.011 | 0.017 | 0.01 | 0.012 |
| Asset index (1 – 10) | 0.041*** | 0.051*** | -0.014*** | -0.012*** | -0.007*** | -0.006*** |
|  | 0.01 | 0.009 | 0.002 | 0.002 | 0.002 | 0.002 |
| Maternal education (years) | 0.011** | 0.013*** | -0.003** | -0.006*** | -0.002** | -0.004*** |
|  | 0.004 | 0.005 | 0.001 | 0.001 | 0.001 | 0.001 |
| Paternal education (years) | 0.005 | 0.006 | -0.002** | -0.001 | -0.002*** | -0.001 |
|  | 0.004 | 0.004 | 0.001 | 0.001 | 0.001 | 0.001 |
| Number of children per woman | -0.014 | -0.015 | 0.002 | 0.002 | 0 | -0.001 |
|  | 0.009 | 0.011 | 0.002 | 0.003 | 0.002 | 0.002 |
| Low birth interval | -0.119** | -0.196*** | 0.023 | 0.074*** | 0.036** | 0.040*** |
|  | 0.057 | 0.071 | 0.016 | 0.018 | 0.015 | 0.015 |
| Male child | -0.232*** | -0.146*** | 0.063*** | 0.033*** | 0.041*** | 0.025*** |
|  | 0.029 | 0.03 | 0.007 | 0.008 | 0.006 | 0.006 |
| Year 2008 | 0.139** | 0.194** | -0.025* | 0.02 | 0.001 | 0.02 |
|  | 0.067 | 0.081 | 0.014 | 0.017 | 0.012 | 0.014 |
| Year 2013 | 0.320*** | 0.382*** | -0.083*** | -0.037 | -0.032* | -0.009 |
|  | 0.085 | 0.107 | 0.019 | 0.023 | 0.017 | 0.019 |
| Year 2018 | 0.139 | 0.185 | -0.062** | -0.028 | -0.060*** | -0.043 |
|  | 0.104 | 0.131 | 0.025 | 0.031 | 0.023 | 0.026 |
| R-squared | 0.198 | 0.192 | 0.167 | 0.155 | 0.134 | 0.115 |
| N | 38028 | 19479 | 38028 | 19479 | 38028 | 19479 |

Note: Clustered robust standard errors are below point estimates. ***, ** and * indicate significance at 1%, 5%, and 10% levels, respectively. The regressions included several time-invariant controls, including zonal fixed effects, dummy variables for practice of Christianity and Islam, month-specific child age dummy variables, and dummy variables for various categories of maternal age and maternal cohort.

**Online Resource 8C: Decomposition of 2003 to 2018 changes in stunting and severe stunting prevalence in urban areas of Nigeria**

|  | Actual change from 2003 to 2018 | **Stunting** | | | **Severe stunting** | | |
| --- | --- | --- | --- | --- | --- | --- | --- |
|  |  | Estimated β | Predicted change | Share of actual change in stunting | Estimated β | Predicted change | Share of actual change in severe stunting |
| Stunting (%) | -0.054 |  | -0.018 | ***31.87*** |  |  |  |
| Severe stunting (%) | -0.053 |  |  |  |  | -0.015 | ***28.15*** |
| Low maternal BMI | -0.030 | 0.062 | -0.002 | 3.45 | 0.038 | -0.001 | 2.16 |
| Maternal height | -0.290 | -0.008 | 0.002 | -4.30 | -0.005 | 0.001 | -2.74 |
| Four or more ANC visits | 0.029 |  |  |  | -0.067 | -0.002 | 3.64 |
| Delivery in health facility | 0.072 | -0.039 | -0.003 | 5.19 | -0.015 | -0.001 | 2.04 |
| Child illness | -0.090 | 0.026 | -0.002 | 4.31 | 0.033 | -0.003 | 5.59 |
| Asset index | -0.207 | -0.015 | 0.003 | -5.75 | -0.007 | 0.001 | -2.74 |
| Maternal education | 2.482 | -0.005 | -0.012 | 22.99 | -0.003 | -0.007 | 14.08 |
| Paternal education | 1.692 | -0.002 | -0.003 | 6.27 | -0.002 | -0.003 | 6.40 |
| Low birth interval | 0.004 | 0.043 | 0.000 | -0.30 | 0.039 | 0.000 | -0.27 |

**Online Resource 9: Age group differences in stunting reduction**

Whereas HAZ did not change among children 6 to 23 months old and improved significantly among children 24 to 59 months old, HAZ deteriorated among children 0 to 6 months old. Stunting reductions were observed among children 6 to 59 months old but there was no change among children 0 to 6 months old (Figure 3, Online Resource 9a). Among nutrition determinants, nutritional status improved among mothers of children 6 to 59 months old, but not among mothers of children 0 to 6 months old. Exclusive breastfeeding increased among children 0 to 6 months old, but complementary feeding declined among children 6 to 23 months old. The prevalence of younger children, (0 to 23 months old) that received the 3 selected health interventions along a continuum of care meaningfully increased, whereas there was no change among children 24 to 59 months old. Still, 29%, 26% and 43% of all children <6, 6 to 23, and 24 to 59 months old, respectively, had received none of the 3 health interventions in 2018 (Online Resource 9a).

Maternal height and ≥4 ANC visits were the only determinants consistently associated with growth outcomes in all 3 age groups. The models identified only a few factors significant for growth outcomes among children <6 months old while many of the determinants were significant for children 24 to 59 months old (Online Resources 9b and 9c).

**Online Resource 9A: Nutrition outcomes and determinants of nutrition in 2003 and 2018 by Child Age Group**

| **Characteristics** | **0 – 5 Months Old** | | | **6 – 23 Months Old** | | | | **24 – 59 Months Old** | | |
| --- | --- | --- | --- | --- | --- | --- | --- | --- | --- | --- |
|  | **2003** | **2018** | **% ∆** | **2003** | **2018** | **% ∆** | **2003** | | **2018** | **% ∆** |
| ***Outcomes*** |  |  |  |  |  |  |  | |  |  |
| Mean HAZ | -0.24 | -0.81 | *240.67**** | -1.57 | -1.40 | *-10.30** | -1.96 | | -1.72 | *-12.44**** |
| % Stunting (HAZ< – 2) | 16.98 | 18.47 | *8.76* | 40.83 | 33.38 | *-18.25**** | 49.22 | | 41.60 | *-15.48**** |
| % Severe Stunting (HAZ< – 3) | 6.85 | 6.43 | *-6.21* | 21.21 | 13.06 | *-38.41**** | 27.24 | | 21.05 | *-22.72**** |
| ***Maternal Nutrition*** |  |  |  |  |  |  |  | |  |  |
| % Underweight (BMI<18.5 kg/m^2^) | 10.51 | 7.88 | *-25.08* | 14.44 | 11.06 | *-23.43*** | 11.44 | | 8.73 | *-23.73*** |
| Average height (cm) | 158.24 | 157.80 | *-0.28* | 158.40 | 158.46 | *0.04* | 158.42 | | 158.52 | *0.06* |
| ***Infant and Young Child Feeding*** |  |  |  |  |  |  |  | |  |  |
| % Exclusive Breastfeeding | 19.94 | 30.98 | *55.37**** |  |  |  |  | |  |  |
| Mean Meal Frequency |  |  |  | 2.32 | 2.30 | *-0.86* |  | |  |  |
| Mean Dietary Diversity |  |  |  | 2.52 | 2.26 | *-10.32**** |  | |  |  |
| ***Health and Health Seeking*** |  |  |  |  |  |  |  | |  |  |
| % ANC at Least Four Visits | 49.33 | 59.81 | *21.24*** | 51.74 | 61.80 | *19.44**** | 51.76 | | 62.14 | *20.05**** |
| % Delivery in Health Facility | 34.34 | 44.53 | *29.68*** | 37.86 | 46.38 | *22.52**** | 36.37 | | 45.31 | *24.59**** |
| % Children Fully Vaccinated at Appropriate Age | 11.61 | 35.93 | *209.46**** | 11.69 | 39.97 | *241.97**** | 11.69 | | 12.78 | *9.33* |
| % Child Illness in 2 Weeks Preceding Survey | 37.20 | 26.18 | *-29.62**** | 57.11 | 44.07 | *-22.84**** | 42.78 | | 34.22 | *-20.01**** |
| ***Water and Sanitation*** |  |  |  |  |  |  |  | |  |  |
| % Piped Drinking Water Source | 13.44 | 12.23 | *-9.02* | 16.28 | 10.51 | *-35.43**** | 15.54 | | 10.73 | *-30.98**** |
| % Borehole/Covered Well Drinking Water Source | 22.76 | 47.12 | *107.05**** | 21.69 | 47.87 | *120.69**** | 21.28 | | 49.03 | *130.44**** |
| % Households with Open Defecation | 21.83 | 22.82 | *4.54* | 24.45 | 23.80 | *-2.67* | 25.17 | | 23.36 | *-7.19* |
| ***Wealth and Education*** |  |  |  |  |  |  |  | |  |  |
| Mean Asset Index | 3.58 | 4.11 | *14.91*** | 3.49 | 4.07 | *16.68**** | 3.54 | | 4.08 | *15.38*** |
| Mean Maternal Education (years) | 4.33 | 6.40 | *47.83**** | 4.30 | 6.44 | *49.86**** | 4.27 | | 6.16 | *44.22**** |
| Mean Paternal Education (years) | 5.61 | 7.60 | *35.48**** | 6.01 | 7.43 | *23.66**** | 5.80 | | 7.37 | *26.95**** |
| ***Demography*** |  |  |  |  |  |  |  | |  |  |
| Mean Number of Children per Woman | 3.85 | 3.86 | *0.21* | 4.06 | 3.86 | *-4.80* | 4.68 | | 4.45 | *-5.07*** |
| % Birth Interval Less than 18 Months | 4.40 | 4.27 | *-2.98* | 5.33 | 4.29 | *-19.58* | 7.34 | | 7.33 | *-0.16* |
| ***Enabling Environment*** |  |  |  |  |  |  |  | |  |  |
| % Received 0 Health Sector Continuum of Care Actions | 45.74 | 29.02 | *-36.56**** | 40.85 | 26.42 | *-35.34**** | 54.18 | | 42.58 | *-21.41**** |
| % Received 3 Health Sector Continuum of Care Actions | 8.04 | 19.16 | *138.37**** | 8.33 | 22.07 | *164.88**** | 4.76 | | 5.31 | *11.64* |
| % Received 0 of 6 Multisectoral Drivers | 6.07 | 3.07 | *-49.46*** | 6.06 | 3.63 | *-40.09*** | 7.58 | | 4.26 | *-43.73**** |
| % Received 6 of 6 Multisectoral Drivers | 4.97 | 9.73 | *95.87* | 4.26 | 11.60 | *172.09**** | 2.32 | | 2.93 | *26.59* |

**Online Resource 9Bi: HAZ and stunting regressions pooled across 2003, 2008, 2013 and 2018 NDHS for children less than 6 (0 to 5) months old**

|  | HAZ | Stunting | Severe stunting |
| --- | --- | --- | --- |
| Low maternal BMI | 0.044 | 0.017 | -0.014 |
|  | 0.138 | 0.03 | 0.018 |
| Maternal height (cm) | 0.032*** | -0.003** | -0.001 |
|  | 0.007 | 0.001 | 0.001 |
| Exclusive breastfeeding | -0.143 | 0.027 | 0.002 |
|  | 0.087 | 0.02 | 0.01 |
| Four or more ANC visits | 0.515** | -0.076** | -0.063*** |
|  | 0.211 | 0.035 | 0.023 |
| Delivery in health facility | 0.052 | -0.004 | -0.011 |
|  | 0.087 | 0.017 | 0.011 |
| Complete age-appropriate vaccinations | 0.069 | -0.013 | -0.007 |
|  | 0.102 | 0.017 | 0.011 |
| Child illness | -0.044 | 0.003 | 0.006 |
|  | 0.095 | 0.019 | 0.013 |
| Piped water | -0.204 | 0.004 | -0.01 |
|  | 0.126 | 0.028 | 0.013 |
| Borehole/covered well water | -0.116 | -0.008 | 0.006 |
|  | 0.087 | 0.016 | 0.011 |
| Open defecation | 0.093 | -0.031 | 0.007 |
|  | 0.124 | 0.025 | 0.016 |
| Asset index (1 – 10) | 0.063*** | -0.008** | -0.002 |
|  | 0.023 | 0.004 | 0.003 |
| Maternal education (years) | -0.011 | -0.001 | -0.001 |
|  | 0.012 | 0.002 | 0.002 |
| Paternal education (years) | -0.01 | 0.001 | 0.002* |
|  | 0.01 | 0.002 | 0.001 |
| Number of children per woman | -0.02 | 0.002 | 0.001 |
|  | 0.023 | 0.004 | 0.003 |
| Low birth interval | 0.11 | -0.038 | -0.021 |
|  | 0.164 | 0.028 | 0.016 |
| Male child | -0.284*** | 0.066*** | 0.036*** |
|  | 0.076 | 0.014 | 0.009 |
| Rural residence | -0.075 | 0.018 | -0.004 |
|  | 0.106 | 0.017 | 0.01 |
| Year 2008 | 0.113 | 0.025 | 0.021 |
|  | 0.163 | 0.032 | 0.023 |
| Year 2013 | 0.471** | -0.054 | -0.005 |
|  | 0.216 | 0.043 | 0.031 |
| Year 2018 | -0.16 | -0.061 | -0.025 |
|  | 0.278 | 0.057 | 0.04 |
| R-squared | 0.083 | 0.071 | 0.06 |
| N | 5699 | 5699 | 5699 |

Note: Clustered robust standard errors are below point estimates. ***, ** and * indicate significance at 1%, 5%, and 10% levels, respectively. The regressions included several time-invariant controls, including zonal fixed effects, dummy variables for practice of Christianity and Islam, month-specific child age dummy variables, and dummy variables for various categories of maternal age and maternal cohort.

**Online Resource 9Bii: HAZ and stunting regressions pooled across 2003, 2008, 2013 and 2018 NDHS for children 6 to 23 months old**

|  | HAZ | Stunting | Severe stunting |
| --- | --- | --- | --- |
| Low maternal BMI | -0.239*** | 0.059*** | 0.031** |
|  | 0.061 | 0.017 | 0.015 |
| Maternal height (cm) | 0.039*** | -0.008*** | -0.006*** |
|  | 0.003 | 0.001 | 0.001 |
| Meal frequency (0 – 7) | 0.019 | -0.004 | -0.003 |
|  | 0.014 | 0.003 | 0.003 |
| Dietary diversity (0 – 6) | 0.034** | -0.007* | -0.003 |
|  | 0.015 | 0.004 | 0.003 |
| Four or more ANC visits | 0.231** | -0.043* | -0.080*** |
|  | 0.116 | 0.025 | 0.021 |
| Delivery in health facility | 0.073 | -0.016 | -0.004 |
|  | 0.052 | 0.014 | 0.01 |
| Complete age-appropriate vaccinations | 0.071 | -0.016 | -0.006 |
|  | 0.048 | 0.012 | 0.009 |
| Child illness | -0.109*** | 0.018 | 0.015* |
|  | 0.041 | 0.011 | 0.009 |
| Piped water | 0.05 | -0.008 | 0.009 |
|  | 0.082 | 0.019 | 0.017 |
| Borehole/covered well water | -0.058 | 0.008 | 0.003 |
|  | 0.045 | 0.011 | 0.009 |
| Open defecation | -0.097 | 0.003 | 0.009 |
|  | 0.084 | 0.017 | 0.015 |
| Asset index (1 – 10) | 0.016 | -0.005* | -0.001 |
|  | 0.013 | 0.003 | 0.002 |
| Maternal education (years) | 0.006 | -0.003 | -0.001 |
|  | 0.006 | 0.002 | 0.001 |
| Paternal education (years) | 0.010** | -0.003** | -0.002** |
|  | 0.005 | 0.001 | 0.001 |
| Number of children per woman | -0.033** | 0.001 | 0 |
|  | 0.013 | 0.003 | 0.003 |
| Low birth interval | 0.048 | -0.005 | 0.005 |
|  | 0.086 | 0.024 | 0.021 |
| Male child | -0.331*** | 0.083*** | 0.069*** |
|  | 0.039 | 0.01 | 0.009 |
| Rural residence | 0.027 | 0.007 | -0.008 |
|  | 0.054 | 0.014 | 0.011 |
| Year 2008 | 0.142* | 0.019 | 0.030* |
|  | 0.082 | 0.02 | 0.017 |
| Year 2013 | 0.541*** | -0.067** | -0.024 |
|  | 0.109 | 0.028 | 0.024 |
| Year 2018 | 0.16 | -0.015 | -0.061* |
|  | 0.147 | 0.04 | 0.036 |
| R-squared | 0.196 | 0.176 | 0.139 |
| N | 16331 | 16331 | 16331 |

Note: Clustered robust standard errors are below point estimates. ***, ** and * indicate significance at 1%, 5%, and 10% levels, respectively. The regressions included several time-invariant controls, including zonal fixed effects, dummy variables for practice of Christianity and Islam, month-specific child age dummy variables, and dummy variables for various categories of maternal age and maternal cohort.

**Online Resource 9Biii: HAZ and stunting regressions pooled across 2003, 2008, 2013 and 2018 NDHS for children 24 to 59 months old**

|  | HAZ | Stunting | Severe stunting |
| --- | --- | --- | --- |
| Low maternal BMI | -0.342*** | 0.078*** | 0.053*** |
|  | 0.047 | 0.014 | 0.012 |
| Maternal height (cm) | 0.036*** | -0.008*** | -0.005*** |
|  | 0.002 | 0.001 | 0.001 |
| Four or more ANC visits | -0.015 | 0.036** | -0.060*** |
|  | 0.079 | 0.018 | 0.016 |
| Delivery in health facility | 0.163*** | -0.054*** | -0.016** |
|  | 0.037 | 0.01 | 0.008 |
| Complete age-appropriate vaccinations | 0.031 | -0.008 | -0.011 |
|  | 0.033 | 0.009 | 0.007 |
| Child illness | -0.207*** | 0.037*** | 0.050*** |
|  | 0.033 | 0.008 | 0.008 |
| Piped water | -0.017 | 0.01 | -0.005 |
|  | 0.046 | 0.013 | 0.012 |
| Borehole/covered well water | 0.009 | -0.011 | -0.008 |
|  | 0.029 | 0.008 | 0.007 |
| Open defecation | -0.160*** | 0.024** | 0.012 |
|  | 0.055 | 0.012 | 0.011 |
| Asset index (1 – 10) | 0.065*** | -0.019*** | -0.009*** |
|  | 0.009 | 0.002 | 0.002 |
| Maternal education (years) | 0.019*** | -0.006*** | -0.005*** |
|  | 0.004 | 0.001 | 0.001 |
| Paternal education (years) | 0.004 | -0.002* | -0.002*** |
|  | 0.003 | 0.001 | 0.001 |
| Number of children per woman | -0.008 | 0.003 | -0.002 |
|  | 0.009 | 0.002 | 0.002 |
| Low birth interval | -0.237*** | 0.062*** | 0.058*** |
|  | 0.056 | 0.016 | 0.015 |
| Male child | -0.122*** | 0.034*** | 0.019*** |
|  | 0.026 | 0.007 | 0.006 |
| Rural residence | -0.101** | 0.034*** | 0.025*** |
|  | 0.041 | 0.009 | 0.008 |
| Year 2008 | 0.178*** | -0.036** | -0.004 |
|  | 0.06 | 0.015 | 0.013 |
| Year 2013 | 0.243*** | -0.073*** | -0.02 |
|  | 0.082 | 0.02 | 0.018 |
| Year 2018 | 0.199** | -0.064** | -0.039* |
|  | 0.097 | 0.026 | 0.023 |
| R-squared | 0.198 | 0.181 | 0.147 |
| N | 33163 | 33163 | 33163 |

Note: Clustered robust standard errors are below point estimates. ***, ** and * indicate significance at 1%, 5%, and 10% levels, respectively. The regressions included several time-invariant controls, including zonal fixed effects, dummy variables for practice of Christianity and Islam, month-specific child age dummy variables, and dummy variables for various categories of maternal age and maternal cohort.

**Online Resource 9Ci: Decomposition of 2003 to 2018 changes in HAZ in children 0 to 5 months old in Nigeria**

|  | Actual change from 2003 to 2018 | Estimated β | Predicted HAZ change |
| --- | --- | --- | --- |
| HAZ | -0.57 |  | 0.01 |
| Maternal height | -0.44 | 0.03 | -0.01 |
| Asset index | 0.53 | 0.04 | 0.02 |

**Online Resource 9Cii: Decomposition of 2003 to 2018 changes in stunting and severe stunting in children 6 to 23 months old in Nigeria**

|  | Actual change | Stunting | | | Severe stunting | | |
| --- | --- | --- | --- | --- | --- | --- | --- |
|  |  | Estimated β | Predicted change | Share of actual change in stunting | Estimated β | Predicted change | Share of actual change in severe stunting |
| Stunting | -0.075 |  | -0.013 | ***16.92*** |  |  |  |
| Severe stunting | -0.081 |  |  |  |  | -0.015 | ***18.74*** |
| Low maternal BMI | -0.034 | 0.061 | -0.002 | 2.77 | 0.034 | -0.001 | 1.41 |
| Maternal height | 0.066 | -0.009 | -0.001 | 0.80 | -0.006 | 0.000 | 0.49 |
| Four or more ANC visits | 0.101 |  |  |  | -0.094 | -0.009 | 11.61 |
| Paternal education | 1.421 | -0.007 | -0.010 | 13.35 | -0.003 | -0.004 | 5.23 |

**Online Resource 9Ciii: Decomposition of 2003 to 2018 changes in HAZ, stunting and severe stunting in children 24 to 59 months old in Nigeria**

|  | Actual change | HAZ | | | Stunting | | | Severe stunting | | |
| --- | --- | --- | --- | --- | --- | --- | --- | --- | --- | --- |
|  |  | Estimated β | Predicted change in means | Share of change in HAZ | Estimated β | Predicted change in means | Share of change in stunting | Estimated β | Predicted change in means | Share of change in severe stunting |
| HAZ | 0.244 |  | 0.126 | ***51.75*** |  |  |  |  |  |  |
| Stunting | -0.076 |  |  |  |  | -0.035 | ***46.10*** |  |  |  |
| Severe stunting | -0.062 |  |  |  |  |  |  |  | -0.033 | ***52.70*** |
| Low maternal BMI | -0.027 | -0.342 | 0.009 | 3.81 | 0.077 | -0.002 | 2.75 | 0.053 | -0.001 | 2.33 |
| Maternal height | 0.100 | 0.036 | 0.004 | 1.47 | -0.008 | -0.001 | 1.05 | -0.005 | 0.000 | 0.80 |
| Four or more ANC visits | 0.104 |  |  |  |  |  |  | -0.066 | -0.007 | 11.07 |
| Delivery in health facility | 0.089 | 0.169 | 0.015 | 6.20 | -0.056 | -0.005 | 6.57 | -0.017 | -0.002 | 2.46 |
| Child illness | -0.086 | -0.207 | 0.018 | 7.27 | 0.037 | -0.003 | 4.16 | 0.050 | -0.004 | 6.92 |
| Open defecation | -0.018 | -0.162 | 0.003 | 1.20 | 0.026 | 0.000 | 0.62 |  |  |  |
| Asset index | 0.544 | 0.066 | 0.036 | 14.73 | -0.019 | -0.010 | 13.58 | -0.010 | -0.005 | 8.79 |
| Maternal education | 1.890 | 0.022 | 0.042 | 17.05 | -0.007 | -0.013 | 17.37 | -0.005 | -0.009 | 15.27 |
| Paternal education | 1.563 |  |  |  |  |  |  | -0.002 | -0.003 | 5.05 |
| Low birth interval | 0.000 | -0.243 | 0.000 | 0.01 | 0.064 | 0.000 | 0.01 | 0.056 | 0.000 | 0.01 |

**Online Resource 10A: Nutrition Outcomes and Determinants of Nutrition among Children 0 to 59 Months Old in 2003 and 2018 by Geopolitical Zone**

| **Characteristics** | **North Central** | | | **North East** | | | **North West** | | | **South East** | | | **South South** | | | | **South West** | | | |
| --- | --- | --- | --- | --- | --- | --- | --- | --- | --- | --- | --- | --- | --- | --- | --- | --- | --- | --- | --- | --- |
|  | **2003** | **2018** | **% ∆** | **2003** | **2018** | **% ∆** | **2003** | **2018** | **% ∆** | **2003** | **2018** | **% ∆** | | **2003** | **2018** | **% ∆** | | **2003** | **2018** | **% ∆** |
| ***Outcomes*** |  |  |  |  |  |  |  |  |  |  |  |  | |  |  |  | |  |  |  |
| Mean HAZ | -1.28 | -1.20 | *6.01* | -1.82 | -1.90 | *-4.15* | -2.37 | -2.21 | *6.65* | -0.66 | -0.84 | *-26.80* | | -0.77 | -0.99 | *-27.77* | | -1.14 | -1.13 | *0.79* |
| % Stunting (HAZ< – 2) | 36.24 | 27.87 | *-23.07**** | 47.40 | 48.71 | *2.76* | 60.21 | 56.79 | *-5.69* | 18.25 | 18.13 | *-0.68* | | 22.54 | 19.88 | *-11.82* | | 28.88 | 22.86 | *-20.82*** |
| % Severe Stunting (HAZ< – 3) | 13.89 | 9.98 | *-28.15**** | 24.86 | 25.58 | *2.89* | 40.11 | 30.67 | *-23.53**** | 5.91 | 5.26 | *-10.98* | | 7.37 | 4.78 | *-35.14** | | 9.69 | 8.17 | *-15.76* |
| ***Maternal Nutrition*** |  |  |  |  |  |  |  |  |  |  |  |  | |  |  |  | |  |  |  |
| % Underweight (BMI<18.5 kg/m^2^) | 6.16 | 6.78 | *10.02* | 21.07 | 19.46 | *-7.66* | 14.71 | 12.43 | *-15.47* | 4.68 | 3.06 | *-34.75* | | 5.10 | 3.28 | *-35.63* | | 8.69 | 5.96 | *-31.39* |
| Average height (cm) | 158.29 | 158.43 | *0.09* | 157.97 | 158.35 | *0.24* | 157.59 | 157.28 | *-0.20* | 159.86 | 159.94 | *0.05* | | 159.13 | 158.40 | *-0.46*** | | 160.05 | 159.31 | *-0.46** |
| ***Health and Health Seeking*** |  |  |  |  |  |  |  |  |  |  |  |  | |  |  |  | |  |  |  |
| % of ANC at Least Four Visits | 57.89 | 56.07 | *-3.14* | 34.33 | 44.35 | *29.19*** | 31.14 | 42.87 | *37.65**** | 84.63 | 83.95 | *-0.80* | | 71.30 | 72.95 | *2.31* | | 95.32 | 89.93 | *-5.66*** |
| % Delivery in Health Facility | 47.02 | 50.27 | *6.89* | 18.20 | 26.18 | *43.88*** | 12.40 | 17.15 | *38.24* | 88.52 | 83.50 | *-5.68* | | 54.30 | 48.91 | *-9.92* | | 78.90 | 75.91 | *-3.79* |
| % Children Fully Vaccinated at Appropriate Age | 14.27 | 22.57 | *58.19*** | 4.65 | 16.27 | *250.12**** | 0.78 | 12.54 | *1500.25**** | 24.41 | 40.32 | *65.15**** | | 15.04 | 32.88 | *118.67**** | | 44.12 | 33.59 | *-23.86**** |
| % Child Illness in 2 Weeks Preceding Survey | 40.05 | 31.37 | *-21.67*** | 63.73 | 54.19 | *-14.97**** | 44.74 | 41.26 | *-7.77* | 38.62 | 32.82 | *-15.01* | | 47.01 | 39.66 | *-15.63* | | 30.28 | 18.76 | *-38.05**** |
| ***Water and Sanitation*** |  |  |  |  |  |  |  |  |  |  |  |  | |  |  |  | |  |  |  |
| % Piped Drinking Water Source | 13.34 | 6.52 | *-51.12*** | 12.14 | 14.31 | *17.87* | 20.56 | 13.32 | *-35.19** | 16.08 | 3.05 | *-81.06**** | | 6.71 | 18.93 | *182.11**** | | 21.88 | 7.93 | *-63.74**** |
| % Borehole/Covered Well Drinking Water Source | 17.77 | 53.04 | *198.51**** | 7.33 | 35.56 | *385.06**** | 15.22 | 41.94 | *175.51**** | 56.59 | 63.46 | *12.15* | | 39.42 | 45.01 | *14.18* | | 33.52 | 57.85 | *72.59**** |
| % Households with Open Defecation | 41.55 | 51.14 | *23.10* | 18.36 | 20.18 | *9.89* | 17.11 | 9.26 | *-45.89*** | 19.77 | 27.00 | *36.56* | | 32.27 | 23.24 | *-27.97* | | 28.58 | 24.83 | *-13.15* |
| ***Wealth and Education*** |  |  |  |  |  |  |  |  |  |  |  |  | |  |  |  | |  |  |  |
| Mean Asset Index | 3.24 | 3.87 | *19.40*** | 2.19 | 2.44 | *11.39* | 3.00 | 2.83 | *-5.58* | 5.82 | 5.18 | *-10.97* | | 4.31 | 5.40 | *25.18*** | | 6.10 | 6.16 | *0.93* |
| Mean Maternal Education (years) | 4.32 | 6.15 | *42.16**** | 2.26 | 3.06 | *35.40** | 2.28 | 2.48 | *8.70* | 8.62 | 10.43 | *21.01** | | 7.32 | 10.09 | *37.87**** | | 8.29 | 10.17 | *22.60**** |
| Mean Paternal Education (years) | 6.94 | 7.81 | *12.62* | 4.29 | 5.16 | *20.40** | 4.29 | 4.67 | *9.01* | 7.83 | 9.56 | *22.04*** | | 7.80 | 10.24 | *31.30**** | | 8.70 | 10.31 | *18.48**** |
| ***Demography*** |  |  |  |  |  |  |  |  |  |  |  |  | |  |  |  | |  |  |  |
| Mean Number of Children per Woman | 4.06 | 3.91 | *-3.77* | 4.82 | 4.70 | *-2.36* | 4.62 | 5.10 | *10.55**** | 4.00 | 3.77 | *-5.63* | | 4.42 | 3.55 | *-19.60**** | | 3.34 | 3.19 | *-4.53* |
| % Birth Interval Less than 18 Months | 4.48 | 5.30 | *18.36* | 7.36 | 6.05 | *-17.85* | 6.43 | 6.10 | *-5.15* | 8.73 | 7.96 | *-8.77* | | 7.08 | 6.24 | *-11.88* | | 3.92 | 4.97 | *26.79* |
| ***Enabling Environment*** |  |  |  |  |  |  |  |  |  |  |  |  | |  |  |  | |  |  |  |
| % Received 0 Health Sector Continuum of Care Actions | 34.69 | 34.69 | *0.00* | 66.86 | 52.40 | *-21.63**** | 73.11 | 58.80 | *-19.57**** | 5.88 | 8.13 | *38.27* | | 30.56 | 28.04 | *-8.25* | | 7.08 | 9.93 | *40.25* |
| % Received 3 Health Sector Continuum of Care Actions | 6.42 | 11.06 | *72.27*** | 2.40 | 5.53 | *130.42**** | 0.39 | 3.28 | *741.03**** | 10.22 | 25.59 | *150.39**** | | 8.64 | 16.43 | *90.16*** | | 27.75 | 21.23 | *-23.50**** |
| % Received 0 of 6 Multisectoral Drivers | 6.96 | 8.19 | *17.67* | 9.56 | 8.29 | *-13.28* | 10.13 | 3.94 | *-61.11*** | 0.76 | 0.38 | *-50.00* | | 1.56 | 0.64 | *-58.97* | | 1.65 | 1.24 | *-24.85* |
| % Received 6 of 6 Multisectoral Drivers | 2.94 | 5.12 | *74.15* | 0.28 | 3.20 | *1042.86**** | 0.22 | 1.98 | *800.00**** | 8.47 | 14.52 | *71.43* | | 5.69 | 8.97 | *57.64* | | 13.41 | 10.33 | *-22.97* |

***, ** and * indicate significance at 1%, 5%, and 10% levels, respectively

**Online Resource 10B: HAZ regressions pooled across 2003, 2008, 2013 and 2018 by geopolitical zone**

|  | NC | NE | NW | SE | SS | SW |
| --- | --- | --- | --- | --- | --- | --- |
| Low maternal BMI | -0.229** | -0.212*** | -0.247*** | -0.482*** | -0.261* | -0.367*** |
|  | 0.102 | 0.07 | 0.062 | 0.131 | 0.149 | 0.086 |
| Maternal height (cm) | 0.048*** | 0.028*** | 0.029*** | 0.050*** | 0.035*** | 0.045*** |
|  | 0.004 | 0.004 | 0.004 | 0.004 | 0.005 | 0.004 |
| Four or more ANC visits | -0.037 | -0.009 | 0.498*** | -0.198 | 0.006 | 0.051 |
|  | 0.135 | 0.166 | 0.13 | 0.218 | 0.176 | 0.191 |
| Delivery in health facility | 0.176*** | 0.117 | 0.107 | 0.106 | 0.169*** | 0.120** |
|  | 0.053 | 0.071 | 0.08 | 0.066 | 0.063 | 0.061 |
| Complete age-appropriate vaccinations | -0.113** | -0.008 | -0.007 | 0.163** | -0.074 | 0.043 |
|  | 0.057 | 0.078 | 0.076 | 0.067 | 0.067 | 0.05 |
| Child illness | -0.048 | -0.295*** | -0.243*** | -0.127 | -0.08 | -0.032 |
|  | 0.056 | 0.052 | 0.045 | 0.078 | 0.065 | 0.053 |
| Piped water | 0.198** | -0.104 | -0.039 | 0.137 | -0.101 | -0.166** |
|  | 0.093 | 0.084 | 0.082 | 0.125 | 0.096 | 0.083 |
| Borehole/covered well water | 0.027 | 0.088 | -0.045 | -0.047 | -0.037 | -0.143*** |
|  | 0.056 | 0.067 | 0.045 | 0.059 | 0.079 | 0.054 |
| Open defecation | -0.222** | 0.087 | -0.107 | 0.174* | -0.260* | -0.13 |
|  | 0.101 | 0.125 | 0.101 | 0.1 | 0.144 | 0.089 |
| Asset index (1 – 10) | 0.045*** | 0.074*** | 0.013 | 0.053*** | 0.035** | 0.076*** |
|  | 0.015 | 0.017 | 0.017 | 0.013 | 0.017 | 0.014 |
| Maternal education (years) | 0.011* | 0.015* | 0.017*** | 0.006 | 0.023** | 0.008 |
|  | 0.006 | 0.008 | 0.006 | 0.009 | 0.011 | 0.006 |
| Paternal education (years) | 0.009** | -0.011 | 0.005 | 0.013* | 0.019* | 0.007 |
|  | 0.005 | 0.007 | 0.005 | 0.007 | 0.01 | 0.006 |
| Number of children per woman | -0.012 | -0.029* | -0.011 | -0.015 | 0.018 | -0.022 |
|  | 0.015 | 0.017 | 0.012 | 0.016 | 0.022 | 0.017 |
| Low birth interval | -0.231** | -0.151* | 0.026 | -0.204** | -0.399*** | -0.138 |
|  | 0.097 | 0.087 | 0.083 | 0.09 | 0.153 | 0.114 |
| Male child | -0.203*** | -0.213*** | -0.257*** | -0.113** | -0.164** | -0.162*** |
|  | 0.045 | 0.042 | 0.043 | 0.048 | 0.075 | 0.043 |
| Rural residence | 0.057 | -0.166* | -0.144** | 0.025 | -0.126* | -0.039 |
|  | 0.074 | 0.086 | 0.073 | 0.066 | 0.072 | 0.069 |
| Year 2008 | -0.235** | -0.035 | 0.644*** | 0.125 | -0.178 | 0.195* |
|  | 0.106 | 0.117 | 0.089 | 0.156 | 0.14 | 0.116 |
| Year 2013 | 0.332** | 0.259 | 0.380*** | 0.324 | 0.493*** | 0.416*** |
|  | 0.141 | 0.159 | 0.115 | 0.197 | 0.188 | 0.146 |
| Year 2018 | 0.144 | -0.055 | 0.350** | 0.134 | 0.09 | 0.18 |
|  | 0.172 | 0.189 | 0.146 | 0.235 | 0.225 | 0.186 |
| R-squared | 0.185 | 0.18 | 0.159 | 0.175 | 0.176 | 0.176 |
| N | 9752 | 11969 | 15306 | 5794 | 6778 | 7908 |

Note: Clustered robust standard errors are below point estimates. ***, ** and * indicate significance at 1%, 5%, and 10% levels, respectively. The regressions included a number of time-invariant controls, including zonal fixed effects, dummy variables for practice of Christianity and Islam, month-specific child age dummy variables, and dummy variables for various categories of maternal age and maternal cohort.

**Online Resource 10C: Stunting regressions pooled across 2003, 2008, 2013 and 2018 by geopolitical zone**

|  | NC | NE | NW | SE | SS | SW |
| --- | --- | --- | --- | --- | --- | --- |
| Low maternal BMI | 0.069** | 0.038** | 0.067*** | 0.109*** | 0.067 | 0.053** |
|  | 0.029 | 0.018 | 0.015 | 0.041 | 0.045 | 0.024 |
| Maternal height (cm) | -0.011*** | -0.006*** | -0.006*** | -0.009*** | -0.008*** | -0.010*** |
|  | 0.001 | 0.001 | 0.001 | 0.001 | 0.001 | 0.001 |
| Four or more ANC visits | 0.090*** | 0.015 | -0.062** | 0.042 | -0.029 | -0.042 |
|  | 0.029 | 0.032 | 0.026 | 0.044 | 0.038 | 0.046 |
| Delivery in health facility | -0.043*** | -0.054*** | -0.017 | -0.039** | -0.046*** | -0.033** |
|  | 0.015 | 0.018 | 0.018 | 0.017 | 0.017 | 0.015 |
| Complete age-appropriate vaccinations | 0.004 | -0.009 | -0.029 | -0.040*** | 0.007 | -0.011 |
|  | 0.016 | 0.021 | 0.018 | 0.013 | 0.015 | 0.013 |
| Child illness | -0.016 | 0.052*** | 0.051*** | 0 | 0.021 | 0.007 |
|  | 0.016 | 0.013 | 0.012 | 0.013 | 0.016 | 0.015 |
| Piped water | -0.03 | 0.003 | 0.022 | -0.051* | 0.023 | 0.015 |
|  | 0.024 | 0.027 | 0.018 | 0.029 | 0.023 | 0.02 |
| Borehole/covered well water | -0.016 | -0.015 | 0.01 | -0.032** | 0.02 | 0.005 |
|  | 0.014 | 0.014 | 0.011 | 0.013 | 0.017 | 0.013 |
| Open defecation | 0.047** | -0.006 | 0.002 | -0.026 | 0.045* | 0.031 |
|  | 0.022 | 0.021 | 0.02 | 0.022 | 0.025 | 0.023 |
| Asset index (0 – 10) | -0.017*** | -0.019*** | -0.007** | -0.007** | -0.013*** | -0.018*** |
|  | 0.004 | 0.004 | 0.004 | 0.003 | 0.004 | 0.004 |
| Maternal education (years) | -0.001 | -0.005** | -0.008*** | -0.003 | -0.003 | -0.005** |
|  | 0.002 | 0.002 | 0.002 | 0.002 | 0.002 | 0.002 |
| Paternal education (years) | -0.004*** | 0.001 | 0 | -0.002 | -0.005** | -0.002 |
|  | 0.002 | 0.002 | 0.001 | 0.002 | 0.002 | 0.002 |
| Number of children per woman | 0.003 | 0.003 | 0.004 | -0.002 | 0 | 0.005 |
|  | 0.004 | 0.004 | 0.003 | 0.004 | 0.004 | 0.005 |
| Low birth interval | 0.05 | 0.04 | -0.016 | 0.058** | 0.082** | 0.079*** |
|  | 0.031 | 0.028 | 0.023 | 0.024 | 0.035 | 0.031 |
| Male child | 0.063*** | 0.049*** | 0.067*** | 0.023* | 0.030** | 0.049*** |
|  | 0.013 | 0.013 | 0.01 | 0.012 | 0.014 | 0.012 |
| Rural residence | 0.007 | 0.049*** | 0.046*** | -0.01 | 0.024 | -0.02 |
|  | 0.017 | 0.018 | 0.016 | 0.013 | 0.016 | 0.017 |
| Year 2008 | 0.033 | 0.035 | -0.125*** | 0.039 | 0.084*** | -0.029 |
|  | 0.026 | 0.024 | 0.021 | 0.027 | 0.027 | 0.029 |
| Year 2013 | -0.102*** | -0.023 | -0.113*** | 0.02 | -0.027 | -0.116*** |
|  | 0.036 | 0.034 | 0.029 | 0.035 | 0.036 | 0.038 |
| Year 2018 | -0.082* | 0.037 | -0.117*** | 0.041 | 0.01 | -0.115** |
|  | 0.046 | 0.046 | 0.039 | 0.046 | 0.048 | 0.05 |
| R-squared | 0.146 | 0.149 | 0.13 | 0.116 | 0.139 | 0.128 |
| N | 9752 | 11969 | 15306 | 5794 | 6778 | 7908 |

Note: Clustered robust standard errors are below point estimates. ***, ** and * indicate significance at 1%, 5%, and 10% levels, respectively. The regressions included a number of time-invariant controls, including zonal fixed effects, dummy variables for practice of Christianity and Islam, month-specific child age dummy variables, and dummy variables for various categories of maternal age and maternal cohort.

**Online Resource 10D: Severe stunting regressions pooled across 2003, 2008, 2013 and 2018 by geopolitical zone**

|  | NC | NE | NW | SE | SS | SW |
| --- | --- | --- | --- | --- | --- | --- |
| Low maternal BMI | 0.002 | 0.051*** | 0.050*** | 0.011 | -0.002 | 0.032* |
|  | 0.022 | 0.016 | 0.015 | 0.022 | 0.023 | 0.019 |
| Maternal height (cm) | -0.006*** | -0.004*** | -0.007*** | -0.003*** | -0.003*** | -0.004*** |
|  | 0.001 | 0.001 | 0.001 | 0.001 | 0.001 | 0.001 |
| Four or more ANC visits | -0.015 | -0.02 | -0.115*** | 0.003 | -0.045* | -0.05 |
|  | 0.023 | 0.028 | 0.025 | 0.029 | 0.025 | 0.033 |
| Delivery in health facility | -0.018 | -0.029* | -0.015 | -0.017 | -0.016 | -0.007 |
|  | 0.011 | 0.016 | 0.017 | 0.011 | 0.011 | 0.01 |
| Complete age-appropriate vaccinations | 0.004 | -0.039** | -0.011 | -0.022*** | -0.004 | -0.008 |
|  | 0.013 | 0.017 | 0.016 | 0.008 | 0.009 | 0.009 |
| Child illness | 0.015 | 0.040*** | 0.074*** | 0.003 | 0.015 | 0 |
|  | 0.012 | 0.012 | 0.012 | 0.008 | 0.01 | 0.01 |
| Piped water | -0.035** | -0.003 | 0.038** | -0.036** | 0.002 | 0.005 |
|  | 0.014 | 0.021 | 0.019 | 0.016 | 0.014 | 0.014 |
| Borehole/covered well water | -0.015 | -0.008 | 0.015 | -0.022*** | -0.002 | -0.002 |
|  | 0.01 | 0.011 | 0.011 | 0.008 | 0.012 | 0.01 |
| Open defecation | 0.025 | -0.004 | 0.02 | -0.022 | 0.008 | 0.023 |
|  | 0.017 | 0.02 | 0.021 | 0.014 | 0.016 | 0.016 |
| Asset index (0 – 10) | -0.009*** | -0.015*** | 0 | -0.006*** | -0.005** | -0.009*** |
|  | 0.003 | 0.004 | 0.003 | 0.002 | 0.002 | 0.003 |
| Maternal education (years) | -0.002 | -0.004** | -0.005*** | -0.002 | -0.003** | -0.002 |
|  | 0.001 | 0.002 | 0.002 | 0.001 | 0.002 | 0.002 |
| Paternal education (years) | -0.003** | 0 | -0.002 | 0 | -0.003* | -0.001 |
|  | 0.001 | 0.001 | 0.001 | 0.001 | 0.001 | 0.001 |
| Number of children per woman | -0.003 | -0.002 | 0.007* | -0.003 | -0.004 | -0.003 |
|  | 0.003 | 0.004 | 0.003 | 0.002 | 0.003 | 0.004 |
| Low birth interval | 0.054* | 0.012 | 0.025 | 0.026* | 0.072** | 0.050** |
|  | 0.028 | 0.026 | 0.023 | 0.014 | 0.03 | 0.022 |
| Male child | 0.020** | 0.055*** | 0.046*** | 0 | 0.035*** | 0.026*** |
|  | 0.01 | 0.011 | 0.01 | 0.007 | 0.009 | 0.008 |
| Rural residence | -0.030** | 0.028* | 0.048*** | -0.016** | 0.002 | 0 |
|  | 0.013 | 0.015 | 0.015 | 0.007 | 0.01 | 0.011 |
| Year 2008 | 0.093*** | 0.072*** | -0.100*** | 0.012 | 0.053*** | 0.024 |
|  | 0.021 | 0.021 | 0.021 | 0.017 | 0.017 | 0.017 |
| Year 2013 | -0.009 | 0.048 | -0.077*** | -0.02 | -0.003 | -0.018 |
|  | 0.028 | 0.03 | 0.029 | 0.021 | 0.022 | 0.024 |
| Year 2018 | -0.043 | 0.076* | -0.153*** | -0.029 | -0.034 | -0.021 |
|  | 0.036 | 0.041 | 0.039 | 0.027 | 0.028 | 0.034 |
| R-squared | 0.095 | 0.107 | 0.109 | 0.057 | 0.096 | 0.082 |
| N | 9752 | 11969 | 15306 | 5794 | 6778 | 7908 |

Note: Clustered robust standard errors are below point estimates. ***, ** and * indicate significance at 1%, 5%, and 10% levels, respectively. The regressions included a number of time-invariant controls, including zonal fixed effects, dummy variables for practice of Christianity and Islam, month-specific child age dummy variables, and dummy variables for various categories of maternal age and maternal cohort.

**Online Resource 10Ei: Decomposition of 2003 to 2018 changes in stunting prevalence in the North Central and South West Geopolitical Zones**

|  | Estimated β | **North Central** | | | **South West** | | |
| --- | --- | --- | --- | --- | --- | --- | --- |
|  |  | Actual change | Predicted stunting change | Share of actual change | Actual change | Predicted stunting change | Share of actual change |
| Stunting |  | -0.084 | -0.024 | ***28.94*** | -0.060 | -0.011 | ***17.60*** |
| Low maternal BMI | 0.062 | 0.006 | 0.000 | -0.46 | -0.027 | -0.002 | 2.81 |
| Maternal height | -0.008 | 0.140 | -0.001 | 1.34 | -0.741 | 0.006 | -9.86 |
| Delivery in health facility | -0.039 | 0.032 | -0.001 | 1.51 | -0.030 | 0.001 | -1.94 |
| Child illness | 0.026 | -0.087 | -0.002 | 2.70 | -0.115 | -0.003 | 4.98 |
| Asset index | -0.015 | 0.629 | -0.009 | 11.28 | 0.057 | -0.001 | 1.42 |
| Maternal education | -0.005 | 1.823 | -0.009 | 10.90 | 1.874 | -0.009 | 15.59 |
| Paternal education | -0.002 | 0.876 | -0.002 | 2.09 | 1.607 | -0.003 | 5.35 |
| Low birth interval | 0.043 | 0.008 | 0.000 | -0.42 | 0.010 | 0.000 | -0.75 |

**Online Resource 10Eii: Decomposition of 2003 to 2018 changes in severe stunting prevalence in the North Central and North West Geopolitical Zones**

|  | Estimated β | **North Central** | | | **North West** | | |
| --- | --- | --- | --- | --- | --- | --- | --- |
|  |  | Actual change | Predicted severe stunting change | Share of actual change | Actual change | Predicted severe stunting change | Share of actual change |
| Severe stunting |  | -0.039 | -0.014 | ***35.54*** | -0.094 | -0.009 | ***9.93*** |
| Low maternal BMI | 0.038 | 0.006 | 0.000 | -0.60 | -0.023 | -0.001 | 0.92 |
| Maternal height | -0.005 | 0.140 | -0.001 | 1.79 | -0.308 | 0.002 | -1.63 |
| Four or more ANC visits | -0.067 | -0.018 | 0.001 | -3.12 | 0.117 | -0.008 | 8.32 |
| Delivery in health | -0.015 | 0.032 | 0.000 | 1.24 | 0.047 | -0.001 | 0.75 |
| Child illness | 0.033 | -0.087 | -0.003 | 7.33 | -0.035 | -0.001 | 1.22 |
| Asset index | -0.007 | 0.629 | -0.004 | 11.25 | -0.167 | 0.001 | -1.24 |
| Maternal education | -0.003 | 1.823 | -0.005 | 13.99 | 0.198 | -0.001 | 0.63 |
| Paternal education | -0.002 | 0.876 | -0.002 | 4.48 | 0.386 | -0.001 | 0.82 |
| Low birth interval | 0.039 | 0.008 | 0.000 | -0.82 | -0.003 | 0.000 | 0.14 |

**Online Resource 11: Nutrition Outcomes and Determinants of Nutrition in Jigawa and Kaduna States 2008 – 2018**

| **Characteristics** | **Jigawa** | | | | | **Kaduna** | | | | |  |
| --- | --- | --- | --- | --- | --- | --- | --- | --- | --- | --- | --- |
|  | **2008** | **2013** | **2018** | **% ∆**  **2008 – 2013** | **% ∆**  **2008 – 2018** | **2008** | **2013** | **2018** | **% ∆**  **2008 – 2013** | **% ∆**  **2008 – 2018** | |
| ***Outcomes*** |  |  |  |  |  |  |  |  |  |  | |
| Mean HAZ | -1.97 | -2.22 | -2.51 | *-12.99* | *-27.39**** | -1.94 | -2.44 | -1.93 | *-25.92**** | *0.49* | |
| % Stunting (HAZ< – 2) | 52.96 | 59.34 | 63.82 | *12.04** | *20.51**** | 52.56 | 56.66 | 49.06 | *7.79* | *-6.67* | |
| % Severe Stunting (HAZ< – 3) | 33.88 | 41.64 | 35.47 | *22.91*** | *4.69* | 33.95 | 41.21 | 21.97 | *21.41*** | *-35.27*** | |
| ***Maternal Nutrition*** |  |  |  |  |  |  |  |  |  |  | |
| % Underweight (BMI<18.5 kg/m^2^) | 18.69 | 17.51 | 18.47 | *-6.31* | *-1.20* | 7.82 | 4.50 | 6.53 | *-42.40** | *-16.51* | |
| Average height (cm) | 156.97 | 156.53 | 156.11 | *-0.29* | *-0.55* | 157.10 | 159.09 | 157.56 | *1.27**** | *0.29* | |
| ***Health and Health Seeking*** |  |  |  |  |  |  |  |  |  |  | |
| % ANC at Least Four Visits | 13.80 | 35.28 | 49.40 | *155.68**** | *258.03**** | 48.97 | 45.63 | 54.96 | *-6.83* | *12.23* | |
| % Delivery in Health Facility | 4.26 | 7.58 | 23.39 | *78.16* | *449.71**** | 19.71 | 32.71 | 18.97 | *65.91** | *-3.74* | |
| % Children Fully Vaccinated at Appropriate Age | 1.63 | 5.25 | 17.78 | *221.81*** | *989.77**** | 18.54 | 29.49 | 13.78 | *59.07* | *-25.66* | |
| % Child Illness in 2 Weeks Preceding Survey | 19.58 | 29.26 | 46.44 | *50.77**** | *137.18**** | 16.70 | 27.04 | 32.85 | *61.92**** | *96.71**** | |
| ***Water and Sanitation*** |  |  |  |  |  |  |  |  |  |  | |
| % Piped Drinking Water Source | 12.57 | 24.72 | 29.57 | *96.73* | *135.32*** | 9.45 | 11.97 | 10.62 | *26.73* | *12.41* | |
| % Borehole/Covered Well Drinking Water Source | 64.90 | 49.08 | 54.78 | *-24.38** | *-15.60* | 35.74 | 49.75 | 54.99 | *39.20** | *53.86*** | |
| % Households with Open Defecation | 31.68 | 24.47 | 16.25 | *-22.75* | *-48.71*** | 16.42 | 16.17 | 4.57 | *-1.49* | *-72.19**** | |
| ***Wealth and Education*** |  |  |  |  |  |  |  |  |  |  | |
| Mean Asset Index | 1.23 | 1.51 | 1.56 | *23.46* | *27.06* | 3.19 | 3.31 | 3.18 | *3.98* | *-0.04* | |
| Mean Maternal Education (years) | 1.19 | 1.15 | 2.07 | *-3.34* | *74.21** | 4.22 | 4.91 | 3.89 | *16.24* | *-7.90* | |
| Mean Paternal Education (years) | 2.86 | 2.82 | 5.86 | *-1.16* | *105.27**** | 6.04 | 6.26 | 5.54 | *3.71* | *-8.19* | |
| ***Demography*** |  |  |  |  |  |  |  |  |  |  | |
| Mean Number of Children per Woman | 5.10 | 4.86 | 5.25 | *-4.59* | *3.05* | 4.38 | 3.87 | 4.65 | *-11.57**** | *6.37* | |
| % Birth Interval Less than 18 Months | 4.12 | 6.91 | 5.54 | *67.88** | *34.57* | 4.54 | 3.50 | 9.60 | *-22.75* | *111.53**** | |
| ***Enabling Environment*** |  |  |  |  |  |  |  |  |  |  | |
| % Received 0 Health Sector Continuum of Care Actions | 88.86 | 73.27 | 57.61 | *-17.54**** | *-35.17**** | 58.94 | 53.21 | 59.07 | *-9.72* | *0.22* | |
| % Received 3 Health Sector Continuum of Care Actions | 0.27 | 0.49 | 0.74 | *81.48* | *174.07* | 4.50 | 14.34 | 1.27 | *218.67**** | *-71.78**** | |
| % Received 0 of 6 Multisectoral Drivers | 7.31 | 7.35 | 1.32 | *0.55* | *-81.94**** | 5.74 | 6.38 | 0.23 | *11.15* | *-95.99**** | |
| % Received 6 of 6 Multisectoral Drivers | 0.18 | 0.30 | 0.59 | *66.67* | *227.78* | 2.9 | 10.7 | 1.01 | *268.97*** | *-65.17** | |

***, ** and * indicate significance at 1%, 5%, and 10% levels, respectively

**Online Resource 12: Scenarios for potential improvements in national level stunting and severe stunting from 2018 to 2025**

|  | Percent/ Means in 2003  A | Percent/ Means in 2018 B | Change in means from 2003 to 2018  (B-A=C) | 2025 Scenario 1: Percent/means continuing past rate of change (B+C=D) | 2025 Scenario 2:  Percent/means of 2018 best GPZ E | Estimated stunting β F | Scenario 1 predicted stunting change  G=(D-B)*F | Scenario 2 predicted stunting change H=(E-B)*F | Estimated severe stunting β I | Scenario 1 predicted severe stunting change  J=(D-B)*I | Scenario 2 predicted severe stunting change K=(E-B)*I |
| --- | --- | --- | --- | --- | --- | --- | --- | --- | --- | --- | --- |
| Stunting | 42.56% | 36.54% | -0.06 |  |  |  | -1.60% | -9.36% |  |  |  |
| Severe Stunting | 22.79% | 16.95% | -0.058 |  |  |  |  |  |  | -1.37% | -7.36% |
| Low maternal BMI | 12.32% | 9.39% | -0.029 | 8.28% | 3.06% | 0.062 | -0.07% | -0.39% | 0.038 | -0.04% | -0.24% |
| Maternal height (cm) | 158.39 | 158.43 | 0.036 | 158.44 | 159.94 | -0.008 | -0.01% | -1.21% | -0.005 | -0.01% | -0.76% |
| Four or more ANC visits | 51.46% | 61.79% | 0.103 | 67.29% | 89.93% |  |  |  | -0.067 | -0.37% | -1.89% |
| Delivery in health facility | 36.61% | 45.58% | 0.09 | 50.48% | 83.50% | -0.039 | -0.19% | -1.48% | -0.015 | -0.07% | -0.57% |
| Child illness | 46.81% | 36.58% | -0.102 | 32.60% | 18.76% | 0.026 | -0.10% | -0.46% | 0.033 | -0.13% | -0.59% |
| Asset index (0 – 10) | 3.53 | 4.08 | 0.555 | 4.37 | 6.16 | -0.015 | -0.43% | -3.12% | -0.007 | -0.20% | -1.45% |
| Maternal education (years) | 4.29 | 6.28 | 1.989 | 7.50 | 10.43 | -0.005 | -0.61% | -2.08% | -0.003 | -0.37% | -1.25% |
| Paternal education (years) | 5.85 | 7.41 | 1.564 | 8.28 | 10.31 | -0.002 | -0.17% | -0.58% | -0.002 | -0.17% | -0.58% |
| Low birth interval | 6.33% | 6.03% | -0.003 | 5.89% | 4.97% | 0.043 | -0.01% | -0.05% | 0.039 | -0.01% | -0.04% |

**Online Resource 13: HAZ regressions for the full national sample by round and pooled model**

|  | **Pooled Model** | **2003** | **2008** | **2013** | **2018** |
| --- | --- | --- | --- | --- | --- |
| Low maternal BMI | -0.261*** | -0.332*** | -0.324*** | -0.223*** | -0.143** |
|  | 0.039 | 0.105 | 0.055 | 0.053 | 0.057 |
| Maternal height (cm) | 0.037*** | 0.031*** | 0.032*** | 0.039*** | 0.043*** |
|  | 0.002 | 0.005 | 0.003 | 0.003 | 0.003 |
| Four or more ANC visits | 0.096 | 0.391** | 0.017 | -0.143 | 0.034 |
|  | 0.069 | 0.184 | 0.12 | 0.126 | 0.097 |
| Delivery in health facility | 0.132*** | 0.196** | 0.091* | 0.024 | 0.117** |
|  | 0.029 | 0.083 | 0.048 | 0.036 | 0.048 |
| Complete age-appropriate vaccinations | 0.02 | 0.158 | 0.054 | 0.156*** | -0.016 |
|  | 0.029 | 0.127 | 0.045 | 0.041 | 0.042 |
| Child illness | -0.157*** | -0.249*** | -0.084** | -0.203*** | -0.081** |
|  | 0.024 | 0.061 | 0.039 | 0.036 | 0.034 |
| Piped water | -0.02 | 0.088 | -0.123* | -0.04 | -0.045 |
|  | 0.042 | 0.098 | 0.074 | 0.069 | 0.057 |
| Borehole/covered well water | -0.024 | -0.06 | -0.068 | 0.024 | -0.034 |
|  | 0.026 | 0.08 | 0.046 | 0.044 | 0.039 |
| Open defecation | -0.120** | -0.192 | -0.128* | -0.146* | 0.084 |
|  | 0.049 | 0.13 | 0.074 | 0.08 | 0.065 |
| Asset index (0 – 10) | 0.051*** | 0.042** | 0.050*** | 0.038*** | 0.055*** |
|  | 0.007 | 0.02 | 0.013 | 0.011 | 0.009 |
| Maternal education (years) | 0.013*** | 0.012 | 0.015*** | 0.008 | 0.017*** |
|  | 0.003 | 0.009 | 0.006 | 0.005 | 0.005 |
| Paternal education (years) | 0.005* | 0.007 | -0.003 | 0.010** | 0.007* |
|  | 0.003 | 0.008 | 0.004 | 0.004 | 0.004 |
| Number of children per woman | -0.017** | 0.007 | -0.002 | -0.034*** | -0.040*** |
|  | 0.007 | 0.02 | 0.012 | 0.011 | 0.011 |
| Low birth interval | -0.148*** | -0.162 | -0.162** | -0.058 | -0.215*** |
|  | 0.045 | 0.121 | 0.072 | 0.079 | 0.065 |
| Male child | -0.201*** | -0.230*** | -0.230*** | -0.203*** | -0.156*** |
|  | 0.022 | 0.068 | 0.032 | 0.028 | 0.03 |
| Rural residence | -0.074** | 0 | -0.200*** | -0.101* | -0.092** |
|  | 0.035 | 0.094 | 0.062 | 0.057 | 0.044 |
| R-squared | 0.209 | 0.291 | 0.146 | 0.235 | 0.263 |
| N | 57507 | 4174 | 18419 | 23835 | 11079 |

**Online Resource 14: Checks for endogenous variables**

**Online Resource 14a: Comparing the baseline models to HAZ, stunting, and severe stunting models with just asset index and parental education included as determinants**

|  | HAZ OLS Full National Sample | HAZ  No Health, No Fertility | Stunting LPM Full National Sample | Stunting  No Health, No Fertility | Severe Stunting LPM Full National Sample | Severe Stunting  No Health, No Fertility |
| --- | --- | --- | --- | --- | --- | --- |
| Low maternal BMI | -0.261*** |  | 0.062*** |  | 0.038*** |  |
|  | 0.039 |  | 0.01 |  | 0.009 |  |
| Maternal height (cm) | 0.037*** |  | -0.008*** |  | -0.005*** |  |
|  | 0.002 |  | 0 |  | 0 |  |
| ≥4 ANC visits | 0.096 |  | 0.007 |  | -0.063*** |  |
|  | 0.069 |  | 0.014 |  | 0.012 |  |
| Delivery in health facility | 0.132*** |  | -0.038*** |  | -0.014** |  |
|  | 0.029 |  | 0.007 |  | 0.006 |  |
| Complete age-appropriate vaccinations | 0.02 |  | -0.011* |  | -0.008 |  |
|  | 0.029 |  | 0.007 |  | 0.005 |  |
| Child illness | -0.157*** |  | 0.026*** |  | 0.033*** |  |
|  | 0.024 |  | 0.006 |  | 0.005 |  |
| Piped water | -0.02 |  | 0.002 |  | 0 |  |
|  | 0.042 |  | 0.01 |  | 0.009 |  |
| Borehole/covered well water | -0.024 |  | -0.005 |  | -0.002 |  |
|  | 0.026 |  | 0.006 |  | 0.005 |  |
| Open defecation | -0.120** |  | 0.014 |  | 0.01 |  |
|  | 0.049 |  | 0.009 |  | 0.008 |  |
| Asset index (1 – 10) | 0.051*** | 0.073*** | -0.014*** | -0.018*** | -0.006*** | -0.010*** |
|  | 0.007 | 0.007 | 0.002 | 0.001 | 0.001 | 0.001 |
| Maternal education (years) | 0.013*** | 0.021*** | -0.004*** | -0.006*** | -0.003*** | -0.004*** |
|  | 0.003 | 0.003 | 0.001 | 0.001 | 0.001 | 0.001 |
| Paternal education (years) | 0.005* | 0.007*** | -0.002** | -0.002*** | -0.002*** | -0.002*** |
|  | 0.003 | 0.003 | 0.001 | 0.001 | 0.001 | 0.001 |
| Number of children per woman | -0.017** |  | 0.003* |  | 0 |  |
|  | 0.007 |  | 0.002 |  | 0.002 |  |
| Low birth interval | -0.148*** |  | 0.041*** |  | 0.039*** |  |
|  | 0.045 |  | 0.012 |  | 0.011 |  |
| Male child | -0.201*** | -0.201*** | 0.052*** | 0.052*** | 0.035*** | 0.035*** |
|  | 0.022 | 0.022 | 0.005 | 0.005 | 0.005 | 0.005 |
| Rural residence | -0.074** | -0.124*** | 0.023*** | 0.030*** | 0.012** | 0.024*** |
|  | 0.035 | 0.035 | 0.007 | 0.007 | 0.006 | 0.005 |
| Year 2008 | 0.170*** | 0.176*** | -0.014 | -0.018* | 0.007 | -0.001 |
|  | 0.054 | 0.055 | 0.011 | 0.011 | 0.01 | 0.01 |
| Year 2013 | 0.354*** | 0.394*** | -0.069*** | -0.082*** | -0.022* | -0.041*** |
|  | 0.069 | 0.069 | 0.015 | 0.014 | 0.013 | 0.013 |
| Year 2018 | 0.162* | 0.159* | -0.053*** | -0.057*** | -0.049*** | -0.064*** |
|  | 0.084 | 0.084 | 0.02 | 0.019 | 0.017 | 0.017 |
| R-squared | 0.209 | 0.191 | 0.177 | 0.165 | 0.139 | 0.13 |
| N | 57507 | 57507 | 57507 | 57507 | 57507 | 57507 |

Note: OLS – ordinary least square model; LPM – linear probability model. Clustered robust standard errors are below point estimates. ***, ** and * indicate significance at 1%, 5%, and 10% levels, respectively. The regressions included several time-invariant controls, including zonal fixed effects, dummy variables for practice of Christianity and Islam, month-specific child age dummy variables, and dummy variables for various categories of maternal age and maternal cohort.

**Online Resource 14B: Decomposition of 2003 to 2018 changes in stunting and severe stunting prevalence in Nigeria based on models with just asset index and parental education included as determinants**

|  | Actual change from 2003 to 2018  (A) | **Stunting** | | | **Severe stunting** | | |
| --- | --- | --- | --- | --- | --- | --- | --- |
|  |  | Estimated β  (B) | Predicted change  (C = A x B) | Predicted change as a share of actual change in stunting  (D=(C/-0.060)*100) | Estimated β  (E) | Predicted change  (F=A x E) | Predicted change as a share of actual change in severe stunting  (G=(F/-0.058)*100) |
| Stunting | -0.060 |  | -0.025 | ***41.66%*** |  |  |  |
| Severe stunting | -0.058 |  |  |  |  | -0.017 | ***28.49%*** |
| Low maternal BMI | -0.029 |  | 0.000 | 0.00% |  | 0.000 | 0.00% |
| Maternal height | 0.036 |  | 0.000 | 0.00% |  | 0.000 | 0.00% |
| Four or more ANC visits | 0.103 |  |  |  |  | 0.000 | 0.00% |
| Delivery in health facility | 0.090 |  | 0.000 | 0.00% |  | 0.000 | 0.00% |
| Child illness | -0.082 |  | 0.000 | 0.00% |  | 0.000 | 0.00% |
| Asset index | 0.555 | -0.018 | -0.010 | 16.61% | -0.010 | -0.006 | 9.50% |
| Maternal education | 1.989 | -0.006 | -0.012 | 19.85% | -0.004 | -0.008 | 13.63% |
| Paternal education | 1.564 | -0.002 | -0.003 | 5.20% | -0.002 | -0.003 | 5.36% |
| Low birth interval | -0.003 |  | 0.000 | 0.00% |  | 0.000 | 0.00% |

**Online Resource 15: Comparison of Linear Probability Models and Logistic regression Models**

**Online Resource 15A: Comparing LPM Models for Stunting and Severe Stunting to Logit Models**

|  | **Stunting LPM Coefficients** | **Stunting Logit Marginal Effects** | **Severe Stunting LPM Coefficients** | **Severe Stunting Logit Marginal Effects** |
| --- | --- | --- | --- | --- |
| Low maternal BMI | 0.062*** | 0.057*** | 0.038*** | 0.030*** |
|  | 0.01 | 0.009 | 0.009 | 0.007 |
| Maternal height (cm) | -0.008*** | -0.008*** | -0.005*** | -0.005*** |
|  | 0 | 0 | 0 | 0 |
| ≥4 ANC visits | 0.007 | 0.021 | -0.063*** | -0.028*** |
|  | 0.014 | 0.013 | 0.012 | 0.011 |
| Delivery in health facility | -0.038*** | -0.037*** | -0.014** | -0.020*** |
|  | 0.007 | 0.007 | 0.006 | 0.006 |
| Complete age-appropriate vaccinations | -0.011* | -0.016** | -0.008 | -0.015** |
|  | 0.007 | 0.007 | 0.005 | 0.007 |
| Child illness | 0.026*** | 0.026*** | 0.033*** | 0.032*** |
|  | 0.006 | 0.006 | 0.005 | 0.005 |
| Piped water | 0.002 | 0.008 | 0 | 0.009 |
|  | 0.01 | 0.01 | 0.009 | 0.009 |
| Borehole/covered well water | -0.005 | -0.004 | -0.002 | -0.001 |
|  | 0.006 | 0.006 | 0.005 | 0.005 |
| Open defecation | 0.014 | 0.017* | 0.01 | 0.018** |
|  | 0.009 | 0.009 | 0.008 | 0.008 |
| Asset index (1 – 10) | -0.014*** | -0.016*** | -0.006*** | -0.009*** |
|  | 0.002 | 0.002 | 0.001 | 0.001 |
| Maternal education (years) | -0.004*** | -0.004*** | -0.003*** | -0.004*** |
|  | 0.001 | 0.001 | 0.001 | 0.001 |
| Paternal education (years) | -0.002** | -0.001* | -0.002*** | -0.001* |
|  | 0.001 | 0.001 | 0.001 | 0.001 |
| Number of children per woman | 0.003* | 0.004** | 0 | 0.001 |
|  | 0.002 | 0.002 | 0.002 | 0.001 |
| Low birth interval | 0.041*** | 0.036*** | 0.039*** | 0.031*** |
|  | 0.012 | 0.012 | 0.011 | 0.01 |
| Male child | 0.052*** | 0.052*** | 0.035*** | 0.036*** |
|  | 0.005 | 0.005 | 0.005 | 0.005 |
| Rural residence | 0.023*** | 0.022*** | 0.012** | 0.012** |
|  | 0.007 | 0.007 | 0.006 | 0.006 |
| Year 2008 | -0.014 | -0.014 | 0.007 | 0.007 |
|  | 0.011 | 0.011 | 0.01 | 0.009 |
| Year 2013 | -0.069*** | -0.071*** | -0.022* | -0.024* |
|  | 0.015 | 0.015 | 0.013 | 0.012 |
| Year 2018 | -0.053*** | -0.055*** | -0.049*** | -0.056*** |
|  | 0.02 | 0.02 | 0.017 | 0.017 |

Note: LPM – linear probability model; Logit – logistic regression model. Clustered robust standard errors are below point estimates. ***, ** and * indicate significance at 1%, 5%, and 10% levels, respectively. The regressions included several time-invariant controls, including zonal fixed effects, dummy variables for practice of Christianity and Islam, month-specific child age dummy variables, and dummy variables for various categories of maternal age and maternal cohort.

**Online Resource 15B: Decomposition of Stunting and Severe Stunting Using Coefficients from Logit Models**

|  | Actual change from 2003 to 2018  (A) | **Stunting** | | | **Severe stunting** | | |
| --- | --- | --- | --- | --- | --- | --- | --- |
|  |  | Estimated β  (B) | Predicted change  (C=A x B) | Predicted change as a share of actual change in stunting  (D = (C/-0.060)*100) | Estimated β  (E) | Predicted change  (F=A x E) | Predicted change as a share of actual change in severe stunting  (G=(F/-0.058)*100) |
| Stunting | -0.060 |  | -0.029 | ***48.99%*** |  |  |  |
| Severe stunting | -0.058 |  |  |  |  | -0.025 | ***42.63%*** |
| Low maternal BMI | -0.029 | 0.057 | -0.002 | 2.77% | 0.030 | -0.001 | 1.50% |
| Maternal height | 0.036 | -0.008 | 0.000 | 0.48% | -0.005 | 0.000 | 0.31% |
| Four or more ANC visits | 0.103 |  |  |  | -0.029 | -0.003 | 5.13% |
| Delivery in health facility | 0.090 | -0.036 | -0.003 | 5.37% | -0.020 | -0.002 | 3.07% |
| Complete age-appropriate vaccinations | 0.123 | -0.016 | -0.002 | 3.27% | -0.016 | -0.002 | 3.37% |
| Child illness | -0.102 | 0.026 | -0.003 | 4.42% | 0.032 | -0.003 | 5.61% |
| Open defecation | -0.011 |  |  |  | 0.019 | 0.000 | 0.35% |
| Asset index (0 – 10) | 0.555 | -0.016 | -0.009 | 14.76% | -0.010 | -0.006 | 9.50% |
| Maternal education (years) | 1.989 | -0.005 | -0.010 | 16.54% | -0.004 | -0.008 | 13.63% |
| Paternal education (years) | 1.564 |  |  |  |  |  |  |
| Number of children per woman | -0.181 | 0.004 | -0.001 | 1.20% |  |  |  |
| Low birth interval | -0.003 | 0.035 | 0.000 | 0.17% | 0.032 | 0.000 | 0.16% |
